# Supplementary material for: Improving reirradiation of recurrent non-small cell lung cancer through non-coplanar beam arrangements
Source: Phys Imaging Radiat Oncol. 2025 Nov 22;36:100874. doi: 10.1016/j.phro.2025.100874 (PMC12701979; doi:10.1016/j.phro.2025.100874)
Supplement: Supplementary Data 1 [file mmc1.docx]

# Supplementary material A Patient characteristics

Table S1 reports the treatment details for the 15 patients considered in this study.

Table S1: Details of the initial and reirradiation treatments for all considered patients. Tumor location is described as ultra-central (UC, when the PTV overlaps with bronchial tree, trachea or esophagus), central (C, when the PTV is at a distance lower than 2 cm from bronchial tree, trachea or esophagus) or peripheral (P, when the PTV is at a distance larger than 2 cm from bronchial tree, trachea or esophagus). Type I reirradiation refers to cases in which there is geometrical overlap between the target volumes in the reirradiation and previous treatments, whereas type II reirradiation describes situations in which there are concerns about cumulative dose toxicity, but no geometrical overlap of the PTV volumes. For patients 1, 3-4, 6, 8-11 and 13, the reirradiation under consideration is the third course of radiotherapy.

|  | **Previous treatment(s)** | | | **Reirradiation treatment** | | | | | |
| --- | --- | --- | --- | --- | --- | --- | --- | --- | --- |
|  | **Tumor location** | **PTV volume (cm^3^)** | **Fractionation scheme** | **Time since previous treatment (months)** | **Reirradiation type** | **Tumor location** | **PTV volume (cm^3^)** | **Fractionation scheme** | **Dose-limiting OARs** |
| **Patient 1** | UC | 288 | 33 x 2 Gy | 15 | I | UC | 49 | 10 x 2.75 Gy | Bronchial tree |
|  | UC | 17 | 10 x 4.5 Gy | 4 |  |  |  |  |  |
| **Patient 2** | P | 98 | 3 x 12.5 Gy | 16 | I | UC | 238 | 30 x 2.2 Gy | Esophagus |
| **Patient 3** | UC | 179 | 30 x 2.2 Gy | 32 | I | UC | 10 | 5 x 5 Gy | Esophagus |
|  | P | 17 | 10 x 4.5 Gy | 6 |  |  |  |  |  |
| **Patient 4** | UC | 432 | 35 x 2 Gy | 19 | I | C | 15 | 10 x 4.5 Gy | Bronchial tree, esophagus, great vessel, trachea |
|  | UC | 66 | 30 x 2 Gy | 17 |  |  |  |  |  |
| **Patient 5** | UC | 603 | 30 x 2 Gy | 4 | II | C | 12 | 5 x 7 Gy | Lungs |
| **Patient 6** | UC | 90 | 33 x 2.2 Gy | 41 | I | UC | 489 | 5 x 4 Gy | Bronchial tree, esophagus,  heart,  great vessel |
|  | UC | 76 | 22 x 2.75 Gy | 29 |  |  |  |  |  |
| **Patient 7** | C | 226 | 30 x 2.2 Gy | 47 | II | P | 20 | 5 x 10 Gy | Thoracic wall |
| **Patient 8** | C | 14 | 20 x 3 Gy | 14 | I | P | 59 | 8 x 5 Gy | Thoracic wall |
|  | P | 11 | 5 x 9 Gy | 13 |  |  |  |  |  |
| **Patient 9** | UC | 525 | 31 x 2 Gy | 61 | I | UC | 288 | 30 x 2 Gy | Brachial plexus, trachea |
|  | C | 17 | 8 x 6 Gy | 42 |  |  |  |  |  |
| **Patient 10** | UC | 525 | 31 x 2 Gy | 8 | I | UC | 12 | 30 x 2 Gy | Bronchial tree, trachea |
|  | P | 17 | 5 x 7 Gy | 8 |  |  |  |  |  |
| **Patient 11** | UC | 149 | 33 x 2 Gy | 6 | II | C | 175 | 20 x 2.75 Gy | Trachea |
|  | P | 14 | 5 x 10 Gy | 6 |  |  |  |  |  |
| **Patient 12** | UC | 1015 | 27 x 2 Gy | 12 | I | UC | 48 | 10 x 4.85 Gy | Bronchial tree |
| **Patient 13** | UC | 372 | 27 x 2.2 Gy | 49 | I | UC | 307 | 10 x 4.5 Gy | Bronchial tree |
|  | C | 113 | 20 x 2.75 Gy | 12 |  |  |  |  |  |
| **Patient 14** | UC | 175 | 19 x 2.75 Gy | 95 | II | C | 155 | 22 x 2.75 Gy | Heart |
| **Patient 15** | UC | 904 | 30 x 2 Gy | 18 | I | UC | 232 | 18 x 2.75 Gy | Bronchial tree, heart |

# Supplementary material B Optimization parameters

In this section, the mathematical formulation of the planning objectives and priorities used in the treatment plan optimization problem are provided for each individual patient, and the dose calculation algorithm which has been used for computing the dose-influence matrices is discussed. The following notation is used:

- $PTV$ = set of voxels belonging to the PTV
- $L$ = set of voxels belonging to the healthy lungs (i.e. excluding the GTV voxels)
- $BT$ = set of voxels belonging to the bronchial tree
- $BT$ = set of voxels belonging to the bronchial tree
- $TW$ = set of voxels belonging to the thoracic wall
- $E$ = set of voxels belonging to the esophagus
- $H$ = set of voxels belonging to the heart
- $SC$ = set of voxels belonging to the spinal cord
- $T$ = set of voxels belonging to the trachea
- $GV$ = set of voxels belonging to the great vessels
- $BPR$ = set of voxels belonging to the right brachial plexus
- $BPL$ = set of voxels belonging to the left brachial plexus
- $TH$ = set of voxels belonging to the thyroid
- $LV$ = set of voxels belonging to the healthy liver (i.e. excluding the GTV voxels)
- $NT$ = set of voxels belonging to the normal tissue (i.e. the entire body except for the PTV)

## B.1 Patient 1

| $f(\boldsymbol{e})$ | = | $\frac{1}{\vert PTV\vert}\sum_{i\in PTV} {[20\left( 58.4-e_{i} \right)}_{+}^{2}+{10\left( e_{i}-65 \right)}_{+}^{2}]$ | $(S1.1)$ |
| --- | --- | --- | --- |
|  | + | $\frac{1}{\vert L\vert}\sum_{i\in L} \left[ 50\tilde{e_{i}}+2000\frac{1}{1+e^{-\left( \tilde{e_{i}}-20 \right)/0.5}}+2000\frac{1}{1+e^{-\left( \tilde{e_{i}}-5 \right)/0.5}} \right]$ | $(S1.2)$ |
|  | + | $\frac{1}{\left\vert BT \right\vert}\sum_{i\in BT} 50\tilde{e_{i}}$ | $(S1.3)$ |
|  | + | $\frac{1}{\left\vert TW \right\vert}\sum_{i\in TW} \left[ 10\tilde{e_{i}}+{0.1\left( \tilde{e_{i}}-74.3 \right)}_{+}^{2} \right]$ | $(S1.4)$ |
|  | + | $\frac{1}{\left\vert E \right\vert}\sum_{i\in E} \left[ 10\tilde{e_{i}}+{0.1\left( \tilde{e_{i}}-114.2 \right)}_{+}^{2} \right]$ | $(S1.5)$ |
|  | + | $\frac{1}{\left\vert H \right\vert}\sum_{i\in H} \left[ 10\tilde{e_{i}}+{0.1\left( \tilde{e_{i}}-32.8 \right)}_{+}^{2} \right]$ | $(S1.6)$ |
|  | + | $\frac{1}{\left\vert SC \right\vert}\sum_{i\in SC} \left[ 10\tilde{e_{i}}+{0.1\left( \tilde{e_{i}}-30.0 \right)}_{+}^{2} \right]$ | $(S1.7)$ |
|  | + | $\frac{1}{\left\vert T \right\vert}\sum_{i\in T} \left[ 10\tilde{e_{i}}+{0.1\left( \tilde{e_{i}}-105.4 \right)}_{+}^{2} \right]$ | $(S1.8)$ |
|  | + | $\frac{1}{\left\vert NT \right\vert}\sum_{i\in NT} {250\left( e_{i}-e_{i}^{max} \right)}_{+}^{2}$ | $(S1.9)$ |

For patient 1, the objective function in Equation (3) reads as follows:

The planning objectives in Equation (S1.2) are used to control the mean EQD2 in the lungs, as well as the volume of healthy lungs exposed to EQD2 values larger than 20 Gy and 5 Gy, respectively. Different than classical dose-volume objectives, which are evaluated using the Heaviside step function $\Theta\left( x \right)$, the objective functions in Equation (S1.2) are evaluated using a continuously differentiable logistic sigmoid function $\zeta_{\epsilon}\left( x \right)=\frac{1}{1+e^{-\left( \tilde{e_{i}}-\tilde{e}_{ref} \right)/\epsilon}}$ (where $\epsilon$ is called smoothness parameter [1]). For $\epsilon\neq0$, a smooth approximation of a dose-volume objective can be defined with a non-vanishing gradient around $e$ = 20 Gy and $e$ = 5 Gy, respectively. In this work, smoothness parameter was set to $\epsilon$ = 0.5 (note that $\zeta_{\epsilon}\left( x \right)\to\Theta\left( x \right)$ for $\epsilon\to0$). The planning objective in Equation (S1.9), instead, corresponds to the normal tissue objective (NTO) implemented in the Eclipse Treatment Planning System (Varian, A Siemens Healtineers Company), where $e_{i}^{max}$ is a voxel-dependent value defined as:

$$e_{i}^{max}= \left\{ \begin{aligned} e_{0} , if x_{i}<x_{0} \\ e_{0}e^{-\kappa\left( x_{i}-x_{0} \right)}+e_{\infty}\left( 1-e^{-\kappa\left( x_{i}-x_{0} \right)} \right) , if x_{i}\geq x_{0} \end{aligned} \right.$$

Here, $x_{i}$ indicates the distance of a normal tissue voxel $i$ from the PTV edge and the NTO parameters were set to $e_{0}=60.0 \mathrm{Gy}_{2}$, $e_{\infty}=12.0 \mathrm{Gy}_{2}$, $x_{0}=0.0 \mathrm{cm}$ and $\kappa=1.0 cm^{-1}$. Note that the planning objectives in Equations (S1.1) and (S1.9) are evaluated only for the EQD2 of the reirradiation plan and not the cumulative EQD2.

## B.2 Patient 2

| $f(\boldsymbol{e})$ | = | $\frac{\omega_{1}}{\vert PTV\vert}\sum_{i\in PTV} {[20\left( 63.5-e_{i} \right)}_{+}^{2}+{10\left( e_{i}-70.7 \right)}_{+}^{2}]$ | $(S2.1)$ |
| --- | --- | --- | --- |
|  | + | $\frac{1}{\vert L\vert}\sum_{i\in L} \left[ 50\tilde{e_{i}}+2000\frac{1}{1+e^{-\left( \tilde{e_{i}}-20 \right)/0.5}}+2000\frac{1}{1+e^{-\left( \tilde{e_{i}}-5 \right)/0.5}} \right]$ | $(S2.2)$ |
|  | + | $\frac{1}{\left\vert E \right\vert}\sum_{i\in E} \left[ 20\tilde{e_{i}}+{0.01\left( \tilde{e_{i}}-17.4 \right)}_{+}^{2} \right]$ | $(S2.3)$ |
|  | + | $\frac{1}{\left\vert H \right\vert}\sum_{i\in H} \left[ 10\tilde{e_{i}}+{10\left( \tilde{e_{i}}-5.3 \right)}_{+}^{2} \right]$ | $(S2.4)$ |
|  | + | $\frac{1}{\left\vert SC \right\vert}\sum_{i\in SC} \left[ 10\tilde{e_{i}}+{70\left( \tilde{e_{i}}-7.1 \right)}_{+}^{2} \right]$ | $(S2.5)$ |
|  | + | $\frac{\omega_{6}}{\left\vert NT \right\vert}\sum_{i\in NT} {250\left( e_{i}-e_{i}^{max} \right)}_{+}^{2}$ | $(S2.6)$ |

For patient 2, the objective function in Equation (3) reads as follows:

where the NTO parameters were set to $e_{0}=64.0 \mathrm{Gy}_{2}$, $e_{\infty}=12.8 \mathrm{Gy}_{2}$, $x_{0}=0.0 \mathrm{cm}$ and $\kappa=1.0 cm^{-1}$. To achieve a similar target coverage and dose conformity in both the coplanar and non-coplanar reirradiation plan, different priorities have been set for the planning objectives in Equations (S2.1) and (S2.6): $\omega_{1}=2.5$ and $\omega_{6}=2$ were set or the coplanar reirradiation plan, and $\omega_{1}=1$ and $\omega_{6}=1$ were set or the non-coplanar reirradiation plan.

## B.3 Patient 3

| $f(\boldsymbol{e})$ | = | $\frac{\omega_{1}}{\vert PTV\vert}\sum_{i\in PTV} {[20\left( 31.25-e_{i} \right)}_{+}^{2}+{10\left( e_{i}-54.7 \right)}_{+}^{2}]$ | $(S3.1)$ |
| --- | --- | --- | --- |
|  | + | $\frac{1}{\vert L\vert}\sum_{i\in L} \left[ 50\tilde{e_{i}}+2000\frac{1}{1+e^{-\left( \tilde{e_{i}}-20 \right)/0.5}}+2000\frac{1}{1+e^{-\left( \tilde{e_{i}}-5 \right)/0.5}} \right]$ | $(S3.2)$ |
|  | + | $\frac{1}{\left\vert E \right\vert}\sum_{i\in E} \left[ \tilde{e_{i}}+\left( \tilde{e_{i}}-88.9 \right)_{+}^{2} \right]$ | $(S3.3)$ |
|  | + | $\frac{1}{\left\vert H \right\vert}\sum_{i\in H} \tilde{e_{i}}$ | $(S3.4)$ |
|  | + | $\frac{1}{\left\vert SC \right\vert}\sum_{i\in SC} \left[ \tilde{e_{i}}+\left( \tilde{e_{i}}-38.4 \right)_{+}^{2} \right]$ | $(S3.5)$ |
|  | + | $\frac{\omega_{6}}{\left\vert NT \right\vert}\sum_{i\in NT} {250\left( e_{i}-e_{i}^{max} \right)}_{+}^{2}$ | $(S3.6)$ |

For patient 3, the objective function in Equation (3) reads as follows:

where the NTO parameters were set to $e_{0}=36.0 \mathrm{Gy}_{2}$, $e_{\infty}=7.2 \mathrm{Gy}_{2}$, $x_{0}=0.0 \mathrm{cm}$ and $\kappa=1.0 cm^{-1}$. Different priorities have been set for the planning objectives in Equations (S3.1) and (S3.6): $\omega_{1}=2.5$ and $\omega_{6}=2$ were set or the coplanar reirradiation plan, and $\omega_{1}=1$ and $\omega_{6}=1$ were set or the non-coplanar reirradiation plan.

## B.4 Patient 4

| $f(\boldsymbol{e})$ | = | $\frac{\omega_{1}}{\vert PTV\vert}\sum_{i\in PTV} {[20\left( 54.4-e_{i} \right)}_{+}^{2}+{10\left( e_{i}-70.2 \right)}_{+}^{2}]$ | $(S4.1)$ |
| --- | --- | --- | --- |
|  | + | $\frac{1}{\vert L\vert}\sum_{i\in L} \left[ 50\tilde{e_{i}}+2000\frac{1}{1+e^{-\left( \tilde{e_{i}}-20 \right)/0.5}}+2000\frac{1}{1+e^{-\left( \tilde{e_{i}}-5 \right)/0.5}} \right]$ | $(S4.2)$ |
|  | + | $\frac{1}{\left\vert BT \right\vert}\sum_{i\in BT} \left[ 10\tilde{e_{i}}+{20\left( \tilde{e_{i}}-108.0 \right)}_{+}^{2} \right]$ | $(S4.3)$ |
|  | + | $\frac{1}{\left\vert E \right\vert}\sum_{i\in E} \left[ 10\tilde{e_{i}}+{20\left( \tilde{e_{i}}-77.7 \right)}_{+}^{2} \right]$ | $(S4.4)$ |
|  | + | $\frac{1}{\left\vert H \right\vert}\sum_{i\in H} \left[ \tilde{e_{i}}+{0.1\left( \tilde{e_{i}}-39.9 \right)}_{+}^{2} \right]$ | $(S4.5)$ |
|  | + | $\frac{1}{\left\vert SC \right\vert}\sum_{i\in SC} \left[ \tilde{e_{i}}+{0.1\left( \tilde{e_{i}}-31.6 \right)}_{+}^{2} \right]$ | $(S4.6)$ |
|  | + | $\frac{1}{\left\vert T \right\vert}\sum_{i\in T} \left[ 10\tilde{e_{i}}+{20\left( \tilde{e_{i}}-105.7 \right)}_{+}^{2} \right]$ | $(S4.7)$ |
|  | + | $\frac{1}{\left\vert GV \right\vert}\sum_{i\in GV} \left[ 10\tilde{e_{i}}+{20\left( \tilde{e_{i}}-103.4 \right)}_{+}^{2} \right]$ | $(S4.8)$ |
|  | + | $\frac{\omega_{9}}{\left\vert NT \right\vert}\sum_{i\in NT} {250\left( e_{i}-e_{i}^{max} \right)}_{+}^{2}$ | $(S4.9)$ |

For patient 4, the objective function in Equation (3) reads as follows:

where the NTO parameters were set to $e_{0}=67.5 \mathrm{Gy}_{2}$, $e_{\infty}=13.5 \mathrm{Gy}_{2}$, $x_{0}=0.0 \mathrm{cm}$ and $\kappa=1.0 cm^{-1}$. Different priorities have been set for the planning objectives in Equations (S4.1) and (S4.9): $\omega_{1}=2.5$ and $\omega_{9}=2$ were set or the coplanar reirradiation plan, and $\omega_{1}=1$ and $\omega_{9}=1$ were set or the non-coplanar reirradiation plan.

## B.5 Patient 5

| $f(\boldsymbol{e})$ | = | $\frac{1}{\vert PTV\vert}\sum_{i\in PTV} {[20\left( 49.6-e_{i} \right)}_{+}^{2}+{10\left( e_{i}-83.3 \right)}_{+}^{2}]$ | $(S5.1)$ |
| --- | --- | --- | --- |
|  | + | $\frac{1}{\vert L\vert}\sum_{i\in L} \left[ 50\tilde{e_{i}}+2000\frac{1}{1+e^{-\left( \tilde{e_{i}}-20 \right)/0.5}}+2000\frac{1}{1+e^{-\left( \tilde{e_{i}}-5 \right)/0.5}} \right]$ | $(S5.2)$ |
|  | + | $\frac{1}{\left\vert BT \right\vert}\sum_{i\in BT} \tilde{e_{i}}$ | $(S5.3)$ |
|  | + | $\frac{1}{\left\vert E \right\vert}\sum_{i\in E} \left[ 20\tilde{e_{i}}+\left( \tilde{e_{i}}-62.0 \right)_{+}^{2} \right]$ | $(S5.4)$ |
|  | + | $\frac{1}{\left\vert H \right\vert}\sum_{i\in H} \tilde{e_{i}}$ | $(S5.5)$ |
|  | + | $\frac{1}{\left\vert SC \right\vert}\sum_{i\in SC} \left[ 20\tilde{e_{i}}+\left( \tilde{e_{i}}-48.8 \right)_{+}^{2} \right]$ | $(S5.6)$ |
|  | + | $\frac{1}{\left\vert T \right\vert}\sum_{i\in T} \left[ 20\tilde{e_{i}}+\left( \tilde{e_{i}}-60.0 \right)_{+}^{2} \right]$ | $(S5.7)$ |
|  | + | $\frac{1}{\left\vert NT \right\vert}\sum_{i\in NT} {250\left( e_{i}-e_{i}^{max} \right)}_{+}^{2}$ | $(S5.8)$ |

For patient 5, the objective function in Equation (3) reads as follows:

where the NTO parameters were set to $e_{0}=70.0 \mathrm{Gy}_{2}$, $e_{\infty}=14.0 \mathrm{Gy}_{2}$, $x_{0}=0.0 \mathrm{cm}$ and $\kappa=1.0 cm^{-1}$.

## B.6 Patient 6

| $f(\boldsymbol{e})$ | = | $\frac{\omega_{1}}{\vert PTV\vert}\sum_{i\in PTV} {[20\left( 21.85-e_{i} \right)}_{+}^{2}+{10\left( e_{i}-24.85 \right)}_{+}^{2}]$ | $(S6.1)$ |
| --- | --- | --- | --- |
|  | + | $\frac{1}{\vert L\vert}\sum_{i\in L} \left[ 50\tilde{e_{i}}+2000\frac{1}{1+e^{-\left( \tilde{e_{i}}-20 \right)/0.5}}+2000\frac{1}{1+e^{-\left( \tilde{e_{i}}-5 \right)/0.5}} \right]$ | $(S6.2)$ |
|  | + | $\frac{1}{\left\vert BT \right\vert}\sum_{i\in BT} 10\tilde{e_{i}}$ | $(S6.3)$ |
|  | + | $\frac{1}{\left\vert E \right\vert}\sum_{i\in E} 10\tilde{e_{i}}$ | $(S6.4)$ |
|  | + | $\frac{1}{\left\vert H \right\vert}\sum_{i\in H} 10\tilde{e_{i}}$ | $(S6.5)$ |
|  | + | $\frac{1}{\left\vert SC \right\vert}\sum_{i\in SC} \left[ 10\tilde{e_{i}}+{10\left( \tilde{e_{i}}-23.9 \right)}_{+}^{2} \right]$ | $(S6.6)$ |
|  | + | $\frac{1}{\left\vert T \right\vert}\sum_{i\in T} 10\tilde{e_{i}}$ | $(S6.7)$ |
|  | + | $\frac{1}{\left\vert GV \right\vert}\sum_{i\in GV} 10\tilde{e_{i}}$ | $(S6.8)$ |
|  | + | $\frac{\omega_{9}}{\left\vert NT \right\vert}\sum_{i\in NT} {250\left( e_{i}-e_{i}^{max} \right)}_{+}^{2}$ | $(S6.9)$ |

For patient 6, the objective function in Equation (3) reads as follows:

where the NTO parameters were set to $e_{0}=25.8 \mathrm{Gy}_{2}$, $e_{\infty}=5.7 \mathrm{Gy}_{2}$, $x_{0}=0.0 \mathrm{cm}$ and $\kappa=1.0 cm^{-1}$. Different priorities have been set for the planning objectives in Equations (S6.1) and (S6.9): $\omega_{1}=2.5$ and $\omega_{9}=2$ were set or the coplanar reirradiation plan, and $\omega_{1}=1$ and $\omega_{9}=1$ were set or the non-coplanar reirradiation plan.

## B.7 Patient 7

| $f(\boldsymbol{e})$ | = | $\frac{\omega_{1}}{\vert PTV\vert}\sum_{i\in PTV} {[20\left( 83.3-e_{i} \right)}_{+}^{2}+{10\left( e_{i}-163.0 \right)}_{+}^{2}]$ | $(S7.1)$ |
| --- | --- | --- | --- |
|  | + | $\frac{1}{\vert L\vert}\sum_{i\in L} \left[ 50\tilde{e_{i}}+2000\frac{1}{1+e^{-\left( \tilde{e_{i}}-20 \right)/0.5}}+2000\frac{1}{1+e^{-\left( \tilde{e_{i}}-5 \right)/0.5}} \right]$ | $(S7.2)$ |
|  | + | $\frac{1}{\left\vert TW \right\vert}\sum_{i\in TW} \left[ 10\tilde{e_{i}}+{10\left( \tilde{e_{i}}-24.4 \right)}_{+}^{2} \right]$ | $(S7.3)$ |
|  | + | $\frac{1}{\left\vert E \right\vert}\sum_{i\in E} \left[ 10\tilde{e_{i}}+{10\left( \tilde{e_{i}}-25.4 \right)}_{+}^{2} \right]$ | $(S7.4)$ |
|  | + | $\frac{1}{\left\vert H \right\vert}\sum_{i\in H} \left[ 10\tilde{e_{i}}+{10\left( \tilde{e_{i}}-64.8 \right)}_{+}^{2} \right]$ | $(S7.5)$ |
|  | + | $\frac{1}{\left\vert SC \right\vert}\sum_{i\in SC} \left[ 10\tilde{e_{i}}+{10\left( \tilde{e_{i}}-8.5 \right)}_{+}^{2} \right]$ | $(S7.6)$ |
|  | + | $\frac{1}{\left\vert T \right\vert}\sum_{i\in T} \left[ 10\tilde{e_{i}}+{10\left( \tilde{e_{i}}-13.2 \right)}_{+}^{2} \right]$ | $(S7.7)$ |
|  | + | $\frac{\omega_{8}}{\left\vert NT \right\vert}\sum_{i\in NT} {250\left( e_{i}-e_{i}^{max} \right)}_{+}^{2}$ | $(S7.8)$ |

For patient 7, the objective function in Equation (3) reads as follows:

where the NTO parameters were set to $e_{0}=130.0 \mathrm{Gy}_{2}$, $e_{\infty}=26.0 \mathrm{Gy}_{2}$, $x_{0}=0.0 \mathrm{cm}$ and $\kappa=1.0 cm^{-1}$. Different priorities have been set for the planning objectives in Equations (S7.1) and (S7.8): $\omega_{1}=2.5$ and $\omega_{8}=2$ were set or the coplanar reirradiation plan, and $\omega_{1}=1$ and $\omega_{8}=1$ were set or the non-coplanar reirradiation plan.

## B.8 Patient 8

| $f(\boldsymbol{e})$ | = | $\frac{1}{\vert PTV\vert}\sum_{i\in PTV} {[20\left( 50.0-e_{i} \right)}_{+}^{2}+{10\left( e_{i}-83.3 \right)}_{+}^{2}]$ | $(S8.1)$ |
| --- | --- | --- | --- |
|  | + | $\frac{1}{\vert L\vert}\sum_{i\in L} \left[ 50\tilde{e_{i}}+2000\frac{1}{1+e^{-\left( \tilde{e_{i}}-20 \right)/0.5}}+2000\frac{1}{1+e^{-\left( \tilde{e_{i}}-5 \right)/0.5}} \right]$ | $(S8.2)$ |
|  | + | $\frac{1}{\left\vert BT \right\vert}\sum_{i\in BT} \tilde{e_{i}}$ | $(S8.3)$ |
|  | + | $\frac{1}{\left\vert TW \right\vert}\sum_{i\in TW} \left[ 10\tilde{e_{i}}+{10\left( \tilde{e_{i}}-214.0 \right)}_{+}^{2} \right]$ | $(S8.4)$ |
|  | + | $\frac{1}{\left\vert E \right\vert}\sum_{i\in E} \tilde{e_{i}}$ | $(S8.5)$ |
|  | + | $\frac{1}{\left\vert H \right\vert}\sum_{i\in H} \tilde{e_{i}}$ | $(S8.6)$ |
|  | + | $\frac{1}{\left\vert SC \right\vert}\sum_{i\in SC} \left[ 10\tilde{e_{i}}+{10\left( \tilde{e_{i}}-11.5 \right)}_{+}^{2} \right]$ | $(S8.7)$ |
|  | + | $\frac{1}{\left\vert T \right\vert}\sum_{i\in T} \left[ \tilde{e_{i}}+{0.1\left( \tilde{e_{i}}-39.9 \right)}_{+}^{2} \right]$ | $(S8.8)$ |
|  | + | $\frac{1}{\left\vert NT \right\vert}\sum_{i\in NT} {250\left( e_{i}-e_{i}^{max} \right)}_{+}^{2}$ | $(S8.9)$ |

For patient 8, the objective function in Equation (3) reads as follows:

where the NTO parameters were set to $e_{0}=64.0 \mathrm{Gy}_{2}$, $e_{\infty}=12.8 \mathrm{Gy}_{2}$, $x_{0}=0.0 \mathrm{cm}$ and $\kappa=1.0 cm^{-1}$.

## B.9 Patient 9

| $f(\boldsymbol{e})$ | = | $\frac{\omega_{1}}{\vert PTV\vert}\sum_{i\in PTV} {[20\left( 58.8-e_{i} \right)}_{+}^{2}+{10\left( e_{i}-61.2 \right)}_{+}^{2}]$ | $(S9.1)$ |
| --- | --- | --- | --- |
|  | + | $\frac{1}{\vert L\vert}\sum_{i\in L} \left[ 50\tilde{e_{i}}+2000\frac{1}{1+e^{-\left( \tilde{e_{i}}-20 \right)/0.5}}+2000\frac{1}{1+e^{-\left( \tilde{e_{i}}-5 \right)/0.5}} \right]$ | $(S9.2)$ |
|  | + | $\frac{1}{\left\vert E \right\vert}\sum_{i\in E} \left[ 10\tilde{e_{i}}+{10\left( \tilde{e_{i}}-75.2 \right)}_{+}^{2} \right]$ | $(S9.3)$ |
|  | + | $\frac{1}{\left\vert H \right\vert}\sum_{i\in H} 0.1\tilde{e_{i}}$ | $(S9.4)$ |
|  | + | $\frac{1}{\left\vert SC \right\vert}\sum_{i\in SC} \left[ 10\tilde{e_{i}}+{10\left( \tilde{e_{i}}-29.9 \right)}_{+}^{2} \right]$ | $(S9.5)$ |
|  | + | $\frac{1}{\left\vert T \right\vert}\sum_{i\in T} \left[ 20\tilde{e_{i}}+{10\left( \tilde{e_{i}}-82.0 \right)}_{+}^{2} \right]$ | $(S9.6)$ |
|  | + | $\frac{1}{\left\vert BPR \right\vert}\sum_{i\in BPR} 10\tilde{e_{i}}$ | $(S9.7)$ |
|  | + | $\frac{\omega_{8}}{\left\vert NT \right\vert}\sum_{i\in NT} {250\left( e_{i}-e_{i}^{max} \right)}_{+}^{2}$ | $(S9.8)$ |

For patient 9, the objective function in Equation (3) reads as follows:

where the NTO parameters were set to $e_{0}=64.0 \mathrm{Gy}_{2}$, $e_{\infty}=12.8 \mathrm{Gy}_{2}$, $x_{0}=0.0 \mathrm{cm}$ and $\kappa=1.0 cm^{-1}$. Different priorities have been set for the planning objectives in Equations (S9.1) and (S9.8): $\omega_{1}=2.5$ and $\omega_{8}=2$ were set or the coplanar reirradiation plan, and $\omega_{1}=1$ and $\omega_{8}=1$ were set or the non-coplanar reirradiation plan.

## B.10 Patient 10

| $f(\boldsymbol{e})$ | = | $\frac{\omega_{1}}{\vert PTV\vert}\sum_{i\in PTV} {[20\left( 58.8-e_{i} \right)}_{+}^{2}+{10\left( e_{i}-61.2 \right)}_{+}^{2}]$ | $(S10.1)$ |
| --- | --- | --- | --- |
|  | + | $\frac{1}{\vert L\vert}\sum_{i\in L} \left[ 50\tilde{e_{i}}+2000\frac{1}{1+e^{-\left( \tilde{e_{i}}-20 \right)/0.5}}+2000\frac{1}{1+e^{-\left( \tilde{e_{i}}-5 \right)/0.5}} \right]$ | $(S10.2)$ |
|  | + | $\frac{1}{\left\vert BT \right\vert}\sum_{i\in BT} 20\tilde{e_{i}}$ | $(S10.3)$ |
|  | + | $\frac{1}{\left\vert TW \right\vert}\sum_{i\in TW} 0.1\tilde{e_{i}}$ | $(S10.4)$ |
|  | + | $\frac{1}{\left\vert E \right\vert}\sum_{i\in E} \left[ 10\tilde{e_{i}}+{10\left( \tilde{e_{i}}-58.6 \right)}_{+}^{2} \right]$ | $(S10.5)$ |
|  | + | $\frac{1}{\left\vert H \right\vert}\sum_{i\in H} 0.1\tilde{e_{i}}$ | $(S10.6)$ |
|  | + | $\frac{1}{\left\vert SC \right\vert}\sum_{i\in SC} \tilde{e_{i}}$ | $(S10.7)$ |
|  | + | $\frac{1}{\left\vert T \right\vert}\sum_{i\in T} 20\tilde{e_{i}}$ | $(S10.8)$ |
|  | + | $\frac{\omega_{8}}{\left\vert NT \right\vert}\sum_{i\in NT} {250\left( e_{i}-e_{i}^{max} \right)}_{+}^{2}$ | $(S10.9)$ |

For patient 10, the objective function in Equation (3) reads as follows:

where the NTO parameters were set to $e_{0}=58.8 \mathrm{Gy}_{2}$, $e_{\infty}=11.8 \mathrm{Gy}_{2}$, $x_{0}=0.0 \mathrm{cm}$ and $\kappa=1.0 cm^{-1}$. Different priorities have been set for the planning objectives in Equations (S10.1) and (S10.9): $\omega_{1}=2.5$ and $\omega_{9}=2$ were set or the coplanar reirradiation plan, and $\omega_{1}=1$ and $\omega_{9}=1$ were set or the non-coplanar reirradiation plan.

## B.11 Patient 11

| $f(\boldsymbol{e})$ | = | $\frac{\omega_{1}}{\vert PTV\vert}\sum_{i\in PTV} {[20\left( 52.1-e_{i} \right)}_{+}^{2}+{10\left( e_{i}-59.7 \right)}_{+}^{2}]$ | $(S11.1)$ |
| --- | --- | --- | --- |
|  | + | $\frac{1}{\left\vert E \right\vert}\sum_{i\in E} \left[ 20\tilde{e_{i}}+{0.1\left( \tilde{e_{i}}-62.5 \right)}_{+}^{2} \right]$ | $(S11.2)$ |
|  | + | $\frac{1}{\left\vert SC \right\vert}\sum_{i\in SC} \left[ 10\tilde{e_{i}}+{10\left( \tilde{e_{i}}-17.1 \right)}_{+}^{2} \right]$ | $(S11.3)$ |
|  | + | $\frac{1}{\left\vert T \right\vert}\sum_{i\in T} \left[ 10\tilde{e_{i}}+\left( \tilde{e_{i}}-62.9 \right)_{+}^{2} \right]$ | $(S11.4)$ |
|  | + | $\frac{1}{\left\vert BPR \right\vert}\sum_{i\in BPR} 10\tilde{e_{i}}$ | $(S11.5)$ |
|  | + | $\frac{1}{\left\vert BPL \right\vert}\sum_{i\in BPL} 10\tilde{e_{i}}$ | $(S11.6)$ |
|  | + | $\frac{1}{\left\vert TH \right\vert}\sum_{i\in TH} \left[ 10\tilde{e_{i}}+0.1\left( \tilde{e_{i}}-0.4 \right)_{+}^{2} \right]$ | $(S11.7)$ |
|  | + | $\frac{\omega_{8}}{\left\vert NT \right\vert}\sum_{i\in NT} {250\left( e_{i}-e_{i}^{max} \right)}_{+}^{2}$ | $(S11.8)$ |

For patient 11, the objective function in Equation (3) reads as follows:

where the NTO parameters were set to $e_{0}=55.0 \mathrm{Gy}_{2}$, $e_{\infty}=11.0 \mathrm{Gy}_{2}$, $x_{0}=0.0 \mathrm{cm}$ and $\kappa=1.0 cm^{-1}$. Different priorities have been set for the planning objectives in Equations (S11.1) and (S11.8): $\omega_{1}=2.5$ and $\omega_{8}=2$ were set or the coplanar reirradiation plan, and $\omega_{1}=1$ and $\omega_{8}=1$ were set or the non-coplanar reirradiation plan.

## B.12 Patient 12

| $f(\boldsymbol{e})$ | = | $\frac{\omega_{1}}{\vert PTV\vert}\sum_{i\in PTV} {[20\left( 59.2-e_{i} \right)}_{+}^{2}+{10\left( e_{i}-60.8 \right)}_{+}^{2}]$ | $(S12.1)$ |
| --- | --- | --- | --- |
|  | + | $\frac{1}{\vert L\vert}\sum_{i\in L} \left[ 50\tilde{e_{i}}+2000\frac{1}{1+e^{-\left( \tilde{e_{i}}-20 \right)/0.5}}+2000\frac{1}{1+e^{-\left( \tilde{e_{i}}-5 \right)/0.5}} \right]$ | $(S12.2)$ |
|  | + | $\frac{1}{\left\vert BT \right\vert}\sum_{i\in BT} \left[ 20\tilde{e_{i}}+\left( \tilde{e_{i}}-52.3 \right)_{+}^{2} \right]$ | $(S12.3)$ |
|  | + | $\frac{1}{\left\vert E \right\vert}\sum_{i\in E} \left[ 10\tilde{e_{i}}+{10\left( \tilde{e_{i}}-52.3 \right)}_{+}^{2} \right]$ | $(S12.4)$ |
|  | + | $\frac{1}{\left\vert H \right\vert}\sum_{i\in H} \left[ 10\tilde{e_{i}}+{10\left( \tilde{e_{i}}-51.6 \right)}_{+}^{2} \right]$ | $(S12.5)$ |
|  | + | $\frac{1}{\left\vert SC \right\vert}\sum_{i\in SC} \left[ 10\tilde{e_{i}}+{10\left( \tilde{e_{i}}-23.8 \right)}_{+}^{2} \right]$ | $(S12.6)$ |
|  | + | $\frac{1}{\left\vert T \right\vert}\sum_{i\in T} \left[ \tilde{e_{i}}+{0.1\left( \tilde{e_{i}}-52.4 \right)}_{+}^{2} \right]$ | $(S12.7)$ |
|  | + | $\frac{1}{\left\vert LV \right\vert}\sum_{i\in LV} \left[ 20\tilde{e_{i}}+{10\left( \tilde{e_{i}}-52.1 \right)}_{+}^{2} \right]$ | $(S12.8)$ |
|  | + | $\frac{\omega_{9}}{\left\vert NT \right\vert}\sum_{i\in NT} {250\left( e_{i}-e_{i}^{max} \right)}_{+}^{2}$ | $(S12.9)$ |

For patient 12, the objective function in Equation (3) reads as follows:

where the NTO parameters were set to $e_{0}=36.0 \mathrm{Gy}_{2}$, $e_{\infty}=7.2 \mathrm{Gy}_{2}$, $x_{0}=0.0 \mathrm{cm}$ and $\kappa=1.0 cm^{-1}$. Different priorities have been set for the planning objectives in Equations (S12.1) and (S12.9): $\omega_{1}=2.5$ and $\omega_{9}=2$ were set or the coplanar reirradiation plan, and $\omega_{1}=1$ and $\omega_{9}=1$ were set or the non-coplanar reirradiation plan.

## B.13 Patient 13

| $f(\boldsymbol{e})$ | = | $\frac{1}{\vert PTV\vert}\sum_{i\in PTV} {[20\left( 52.8-e_{i} \right)}_{+}^{2}+{10\left( e_{i}-56.0 \right)}_{+}^{2}]$ | $(S13.1)$ |
| --- | --- | --- | --- |
|  | + | $\frac{1}{\vert L\vert}\sum_{i\in L} \left[ 50\tilde{e_{i}}+2000\frac{1}{1+e^{-\left( \tilde{e_{i}}-20 \right)/0.5}}+2000\frac{1}{1+e^{-\left( \tilde{e_{i}}-5 \right)/0.5}} \right]$ | $(S13.2)$ |
|  | + | $\frac{1}{\left\vert BT \right\vert}\sum_{i\in BT} \left[ 10\tilde{e_{i}}+10\left( \tilde{e_{i}}-115.3 \right)_{+}^{2} \right]$ | $(S13.3)$ |
|  | + | $\frac{1}{\left\vert TW \right\vert}\sum_{i\in TW} \left[ 10\tilde{e_{i}}+10\left( \tilde{e_{i}}-60.8 \right)_{+}^{2} \right]$ | $(S13.4)$ |
|  | + | $\frac{1}{\left\vert E \right\vert}\sum_{i\in E} \left[ 10\tilde{e_{i}}+{10\left( \tilde{e_{i}}-71.3 \right)}_{+}^{2} \right]$ | $(S13.5)$ |
|  | + | $\frac{1}{\left\vert H \right\vert}\sum_{i\in H} \left[ \tilde{e_{i}}+\left( \tilde{e_{i}}-60.0 \right)_{+}^{2} \right]$ | $(S13.6)$ |
|  | + | $\frac{1}{\left\vert SC \right\vert}\sum_{i\in SC} \left[ 10\tilde{e_{i}}+{10\left( \tilde{e_{i}}-22.4 \right)}_{+}^{2} \right]$ | $(S13.7)$ |
|  | + | $\frac{1}{\left\vert T \right\vert}\sum_{i\in T} \left[ 10\tilde{e_{i}}+{10\left( \tilde{e_{i}}-80.4 \right)}_{+}^{2} \right]$ | $(S13.8)$ |
|  | + | $\frac{1}{\left\vert BPR \right\vert}\sum_{i\in BPR} \left[ \tilde{e_{i}}+\left( \tilde{e_{i}}-6.2 \right)_{+}^{2} \right]$ | $(S13.9)$ |
|  | + | $\frac{1}{\left\vert NT \right\vert}\sum_{i\in NT} {250\left( e_{i}-e_{i}^{max} \right)}_{+}^{2}$ | $(S13.10)$ |

For patient 13, the objective function in Equation (3) reads as follows:

where the NTO parameters were set to $e_{0}=74.9 \mathrm{Gy}_{2}$, $e_{\infty}=15.0 \mathrm{Gy}_{2}$, $x_{0}=0.0 \mathrm{cm}$ and $\kappa=1.0 cm^{-1}$.

## B.14 Patient 14

| $f(\boldsymbol{e})$ | = | $\frac{\omega_{1}}{\vert PTV\vert}\sum_{i\in PTV} {[20\left( 63.6-e_{i} \right)}_{+}^{2}+{10\left( e_{i}-64.9 \right)}_{+}^{2}]$ | $(S14.1)$ |
| --- | --- | --- | --- |
|  | + | $\frac{1}{\vert L\vert}\sum_{i\in L} \left[ 50\tilde{e_{i}}+2000\frac{1}{1+e^{-\left( \tilde{e_{i}}-20 \right)/0.5}}+2000\frac{1}{1+e^{-\left( \tilde{e_{i}}-5 \right)/0.5}} \right]$ | $(S14.2)$ |
|  | + | $\frac{1}{\left\vert E \right\vert}\sum_{i\in E} \left[ 10\tilde{e_{i}}+{10\left( \tilde{e_{i}}-56.7 \right)}_{+}^{2} \right]$ | $(S14.3)$ |
|  | + | $\frac{1}{\left\vert H \right\vert}\sum_{i\in H} 20\tilde{e_{i}}$ | $(S14.4)$ |
|  | + | $\frac{1}{\left\vert SC \right\vert}\sum_{i\in SC} \left[ 10\tilde{e_{i}}+{10\left( \tilde{e_{i}}-18.9 \right)}_{+}^{2} \right]$ | $(S14.5)$ |
|  | + | $\frac{\omega_{6}}{\left\vert NT \right\vert}\sum_{i\in NT} {250\left( e_{i}-e_{i}^{max} \right)}_{+}^{2}$ | $(S14.6)$ |

For patient 14, the objective function in Equation (3) reads as follows:

where the NTO parameters were set to $e_{0}=68.7 \mathrm{Gy}_{2}$, $e_{\infty}=13.7 \mathrm{Gy}_{2}$, $x_{0}=0.0 \mathrm{cm}$ and $\kappa=1.0 cm^{-1}$. Different priorities have been set for the planning objectives in Equations (S14.1) and (S14.6): $\omega_{1}=2.5$ and $\omega_{6}=2$ were set or the coplanar reirradiation plan, and $\omega_{1}=1$ and $\omega_{6}=1$ were set or the non-coplanar reirradiation plan.

## B.15 Patient 15

| $f(\boldsymbol{e})$ | = | $\frac{1}{\vert PTV\vert}\sum_{i\in PTV} {[20\left( 51.9-e_{i} \right)}_{+}^{2}+{10\left( e_{i}-53.2 \right)}_{+}^{2}]$ | $(S15.1)$ |
| --- | --- | --- | --- |
|  | + | $\frac{1}{\vert L\vert}\sum_{i\in L} \left[ 50\tilde{e_{i}}+2000\frac{1}{1+e^{-\left( \tilde{e_{i}}-20 \right)/0.5}}+2000\frac{1}{1+e^{-\left( \tilde{e_{i}}-5 \right)/0.5}} \right]$ | $(S15.2)$ |
|  | + | $\frac{1}{\left\vert BT \right\vert}\sum_{i\in BT} 10\tilde{e_{i}}$ | $(S15.3)$ |
|  | + | $\frac{1}{\left\vert E \right\vert}\sum_{i\in E} \left[ 10\tilde{e_{i}}+\left( \tilde{e_{i}}-18.7 \right)_{+}^{2} \right]$ | $(S15.4)$ |
|  | + | $\frac{1}{\left\vert H \right\vert}\sum_{i\in H} \left[ 20\tilde{e_{i}}+{20\left( \tilde{e_{i}}-59.4 \right)}_{+}^{2} \right]$ | $(S15.5)$ |
|  | + | $\frac{1}{\left\vert SC \right\vert}\sum_{i\in SC} \left[ 10\tilde{e_{i}}+{10\left( \tilde{e_{i}}-38.2 \right)}_{+}^{2} \right]$ | $(S15.6)$ |
|  | + | $\frac{1}{\left\vert T \right\vert}\sum_{i\in T} \left[ \tilde{e_{i}}+{0.1\left( \tilde{e_{i}}-58.8 \right)}_{+}^{2} \right]$ | $(S15.7)$ |
|  | + | $\frac{1}{\left\vert NT \right\vert}\sum_{i\in NT} {250\left( e_{i}-e_{i}^{max} \right)}_{+}^{2}$ | $(S15.8)$ |

For patient 15, the objective function in Equation (3) reads as follows:

where the NTO parameters were set to $e_{0}=56.1 \mathrm{Gy}_{2}$, $e_{\infty}=11.2 \mathrm{Gy}_{2}$, $x_{0}=0.0 \mathrm{cm}$ and $\kappa=1.0 cm^{-1}$.

## B.16 Dose calculation algorithm

Calculation of the dose-influence matrix is performed with the open-source radiotherapy planning research platform CERR [2], using a quadrant infinite beam (QIB) algorithm [3]. The bixel size is set to 5 x 5 mm^2^ and the photon energy is 6 MV for all candidate beam orientations. For each patient, a non-uniform dose grid size is used throughout the body: the original voxel resolution is used in the PTV and close to the PTV, where a larger dose gradient is expected. At a distance between 2 cm and 4 cm from the PTV edge, medium-size voxels are used with 8-fold volume, whereas at distances larger than 4 cm from the PTV edge large-size voxels are used with 64-fold volume. As previously shown by Mueller *et al* [4], the use of a non-uniform dose grid size allows to considerably enhance the computational efficiency with negligible trade-offs on the plan accuracy. All dosimetric results, however, are evaluated based on the finest (small) dose grid size.

## References Supplementary material B

[1] Andersson B (2017). Mathematical optimization of radiation therapy goal fulfillment. MSc thesis. Uppsala University.

[2] Deasy JO, Blanco AI, and Clark VH. CERR: a computational environment for radiotherapy research. Medical Physics, 30:979, 2003.

[3] Kalinin ED, J. A method for fast 3-D IMRT dose calculations the quadrant infinite beam (QIB) algorithm. 45^th^ annual meeting of the American Association of Physicist in medicine. 2003.

[4] Mueller S, Guyer G, Volken W, et al. Efficiency enhancements of a Monte Carlo beamlet based treatment planning process: implementation and parameter study. Phys Med Biol. 2023;68(4):10.1088/1361-6560/acb480. Published 2023 Feb 13. doi:10.1088/1361-6560/acb480.

# Supplementary material C Results

In this section, the dosimetric results and the dose distributions obtained using both the coplanar and non-coplanar reirradiation plans are reported for each individual patient.

## C.1 Patient 1

The dose distributions along with the corresponding gantry-couch paths obtained for patient 1 are reported in Figure S1 for both the coplanar and non-coplanar reirradiation plans. The dosimetric results are instead detailed in Table S2.


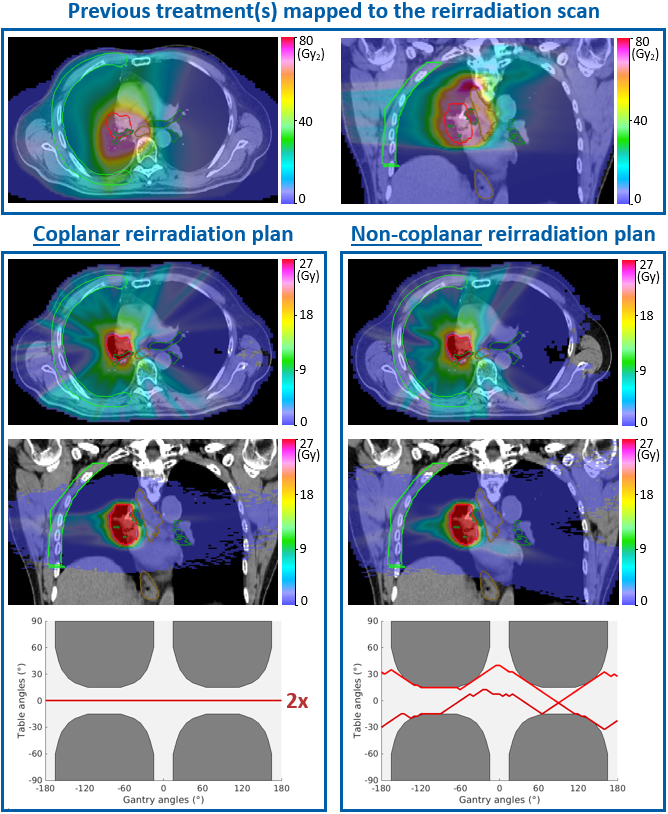


Figure S1: Comparison of the coplanar and non-coplanar reirradiation plans generated for patient 1. Contours of PTV (red), bronchial tree (dark green), esophagus (brown) and thoracic wall (light green) are delineated in both the transversal and coronal planes of the reirradiation scan. The gantry-couch paths for both reirradiation plans are also shown, where dark grey regions indicate beam orientations leading to collision between gantry and couch (and are therefore excluded from the set of candidate beam orientations).

Table S2: Cumulative EQD2 metrics for patient 1 achieved both prior to reirradiation and after reirradiation using the coplanar and non-coplanar plans. Percentage differences in dosimetric values between the coplanar and non-coplanar reirradiation plans are reported for each parameter. The conformity index in the PTV is given by $CI=\frac{\left( V_{PTV}\cap V_{d_{pres}} \right)^{2}}{V_{PTV} V_{d_{pres}}}$ (where $V_{PTV}$ is the PTV volume and $V_{d_{pres}}$ is the total volume receiving the prescribed cumulative dose $d_{pres}$), while the homogeneity index is expressed as $HI=\frac{d_{2}-d_{98}}{d_{pres}}$ (where $d_{2}$ and $d_{98}$ are the doses received by 2% and 98% of the PTV volume in the reirradiation plan, respectively).

| **OAR** | **EQD2 parameter** | **Previous treatment(s)** | **Coplanar reirradiation plan** | **Non-coplanar reirradiation plan** |
| --- | --- | --- | --- | --- |
| Bronchial tree^#^ | D_max_ (Gy) | 71.4 | 124.0 | 122.5 (-1.2%) |
|  | D_mean_ (Gy) | 37.8 | 40.4 | 40.2 (-0.5%) |
| Esophagus | D_max_ (Gy) | 126.9 | 126.9 | 126.9 (=) |
|  | D_mean_ (Gy) | 35.5 | 37.0 | 36.8 (-0.5%) |
| Heart | D_max_ (Gy) | 36.5 | 45.0 | 49.2 (+9.3%) |
|  | D_mean_ (Gy) | 1.51 | 1.60 | 1.98 (+23.8%) |
| Spinal cord | D_max_ (Gy) | 33.3 | 36.0 | 33.9 (-5.8%) |
|  | D_mean_ (Gy) | 9.99 | 11.0 | 10.9 (-0.9%) |
| Thoracic wall | D_max_ (Gy) | 82.6 | 82.6 | 82.6 (=) |
|  | D_mean_ (Gy) | 12.6 | 15.1 | 14.9 (-1.3%) |
| Trachea | D_max_ (Gy) | 117.2 | 117.2 | 117.2 (=) |
|  | D_mean_ (Gy) | 45.7 | 45.8 | 45.8 (=) |
| Lungs-GTV | D_mean_ (Gy) | 11.7 | 14.5 | 14.4 (-0.7%) |
|  | V_5Gy_ (%) | 47.9 | 49.2 | 48.9 (-0.6%) |
|  | V_20Gy_ (%) | 19.5 | 23.1 | 22.1 (-4.3%) |
| PTV | HI | - | 0.18 | 0.17 (-5.6%) |
|  | CI | - | 0.73 | 0.73 (=) |

^#^ OARs which overlap with the PTV

## C.2 Patient 2

The dose distributions along with the corresponding gantry-couch paths obtained for patient 2 are reported in Figure S2 for both the coplanar and non-coplanar reirradiation plans. The dosimetric results are instead detailed in Table S3.


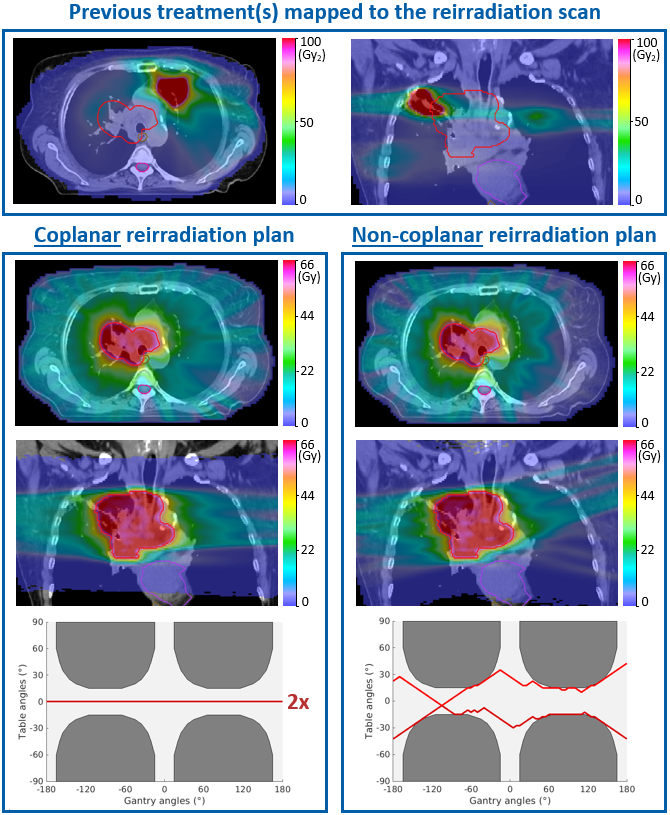


Figure S2: Comparison of the coplanar and non-coplanar reirradiation plans generated for patient 2. Contours of PTV (red), spinal cord (pink), esophagus (brown) and heart (violet) are delineated in both the transversal and coronal planes of the reirradiation scan. The gantry-couch paths for both reirradiation plans are also shown, where dark grey regions indicate beam orientations leading to collision between gantry and couch (and are therefore excluded from the set of candidate beam orientations).

Table S3: Cumulative EQD2 metrics for patient 2 achieved both prior to reirradiation and after reirradiation using the coplanar and non-coplanar plans. Percentage differences in dosimetric values between the coplanar and non-coplanar reirradiation plans are reported for each parameter.

| **OAR** | **EQD2 parameter** | **Previous treatment(s)** | **Coplanar reirradiation plan** | **Non-coplanar reirradiation plan** |
| --- | --- | --- | --- | --- |
| Esophagus^#^ | D_max_ (Gy) | 19.3 | 63.8 | 58.0 (-9.1%) |
|  | D_mean_ (Gy) | 4.05 | 13.1 | 9.79 (-25.3%) |
| Heart | D_max_ (Gy) | 5.89 | 18.3 | 11.9 (-35.0%) |
|  | D_mean_ (Gy) | 0.90 | 1.08 | 1.07 (-0.9%) |
| Spinal cord | D_max_ (Gy) | 7.87 | 12.1 | 10.0 (-17.4%) |
|  | D_mean_ (Gy) | 3.12 | 5.41 | 4.85 (-10.4%) |
| Lungs-GTV | D_mean_ (Gy) | 9.37 | 16.0 | 15.5 (-3.1%) |
|  | V_5Gy_ (%) | 30.4 | 43.8 | 42.9 (-2.1%) |
|  | V_20Gy_ (%) | 9.62 | 24.6 | 21.9 (-11.0%) |
| PTV | HI | - | 0.18 | 0.20 (+11.1%) |
|  | CI | - | 0.31 | 0.32 (+3.2) |

^#^ OARs which overlap with the PTV

## C.3 Patient 3

The dose distributions along with the corresponding gantry-couch paths obtained for patient 3 are reported in Figure S3 for both the coplanar and non-coplanar reirradiation plans. The dosimetric results are instead detailed in Table S4.


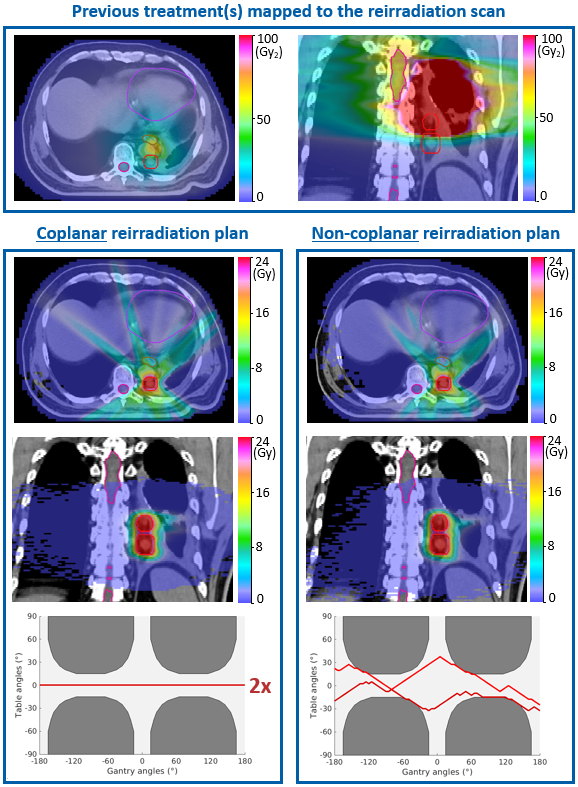


Figure S3: Comparison of the coplanar and non-coplanar reirradiation plans generated for patient 3. Contours of PTV (red), spinal cord (pink), esophagus (brown) and heart (violet) are delineated in both the transversal and coronal planes of the reirradiation scan. The gantry-couch paths for both reirradiation plans are also shown, where dark grey regions indicate beam orientations leading to collision between gantry and couch (and are therefore excluded from the set of candidate beam orientations).

Table S4: Cumulative EQD2 metrics for patient 3 achieved both prior to reirradiation and after reirradiation using the coplanar and non-coplanar plans. Percentage differences in dosimetric values between the coplanar and non-coplanar reirradiation plans are reported for each parameter.

| **OAR** | **EQD2 parameter** | **Previous treatment(s)** | **Coplanar reirradiation plan** | **Non-coplanar reirradiation plan** |
| --- | --- | --- | --- | --- |
| Esophagus | D_max_ (Gy) | 98.8 | 100.6 | 99.7 (-0.9%) |
|  | D_mean_ (Gy) | 36.7 | 38.1 | 37.5 (-1.6%) |
| Heart | D_max_ (Gy) | 82.5 | 83.2 | 82.8 (-0.5%) |
|  | D_mean_ (Gy) | 22.6 | 23.4 | 23.0 (-1.7%) |
| Spinal cord | D_max_ (Gy) | 42.7 | 42.8 | 42.8 (=) |
|  | D_mean_ (Gy) | 13.4 | 13.5 | 13.5 (=) |
| Lungs-GTV | D_mean_ (Gy) | 18.1 | 18.2 | 18.1 (-0.5%) |
|  | V_5Gy_ (%) | 91.4 | 91.5 | 91.5 (=) |
|  | V_20Gy_ (%) | 29.3 | 29.4 | 29.3 (-0.3%) |
| PTV | HI | - | 0.42 | 0.42 (=) |
|  | CI | - | 0.82 | 0.85 (+3.7) |

## C.4 Patient 4

The dose distributions along with the corresponding gantry-couch paths obtained for patient 4 are reported in Figure 3 of the main manuscript for both the coplanar and non-coplanar reirradiation plans. The dosimetric results are instead detailed in Table S5.

Table S5: Cumulative EQD2 metrics for patient 4 achieved both prior to reirradiation and after reirradiation using the coplanar and non-coplanar plans. Percentage differences in dosimetric values between the coplanar and non-coplanar reirradiation plans are reported for each parameter.

| **OAR** | **EQD2 parameter** | **Previous treatment(s)** | **Coplanar reirradiation plan** | **Non-coplanar reirradiation plan** |
| --- | --- | --- | --- | --- |
| Bronchial tree | D_max_ (Gy) | 120.0 | 141.3 | 132.3 (-6.4%) |
|  | D_mean_ (Gy) | 89.3 | 94.1 | 93.3 (-0.9%) |
| Esophagus | D_max_ (Gy) | 86.3 | 95.5 | 90.9 (-5.2%) |
|  | D_mean_ (Gy) | 41.5 | 42.1 | 41.9 (-0.5%) |
| Heart | D_max_ (Gy) | 44.4 | 44.4 | 44.4 (=) |
|  | D_mean_ (Gy) | 2.14 | 2.14 | 2.14 (=) |
| Spinal cord | D_max_ (Gy) | 35.1 | 45.1 | 41.2 (-8.6%) |
|  | D_mean_ (Gy) | 9.79 | 10.7 | 10.7 (=) |
| Great vessel | D_max_ (Gy) | 114.9 | 120.6 | 115.0 (-4.6%) |
|  | D_mean_ (Gy) | 42.8 | 43.7 | 43.5 (-0.5%) |
| Trachea | D_max_ (Gy) | 117.5 | 127.6 | 122.7 (-3.8%) |
|  | D_mean_ (Gy) | 51.7 | 52.7 | 52.5 (-0.4%) |
| Lungs-GTV | D_mean_ (Gy) | 11.1 | 12.2 | 12.1 (-0.8%) |
|  | V_5Gy_ (%) | 45.0 | 45.5 | 45.3 (-0.4%) |
|  | V_20Gy_ (%) | 17.4 | 18.8 | 18.7 (-0.5%) |
| PTV | HI | - | 0.29 | 0.31 (+6.9%) |
|  | CI | - | 0.75 | 0.75 (=) |

## C.5 Patient 5

The dose distributions along with the corresponding gantry-couch paths obtained for patient 5 are reported in Figure S4 for both the coplanar and non-coplanar reirradiation plans. The dosimetric results are instead detailed in Table S6.


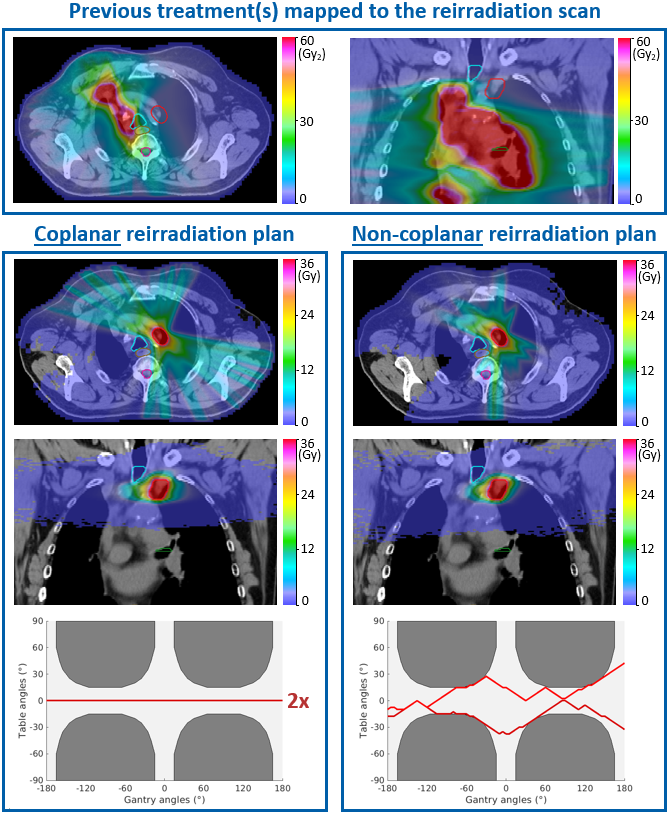


Figure S4: Comparison of the coplanar and non-coplanar reirradiation plans generated for patient 5. Contours of PTV (red), spinal cord (pink), esophagus (brown) and trachea (light blue) are delineated in both the transversal and coronal planes of the reirradiation scan. The gantry-couch paths for both reirradiation plans are also shown, where dark grey regions indicate beam orientations leading to collision between gantry and couch (and are therefore excluded from the set of candidate beam orientations).

Table S6: Cumulative EQD2 metrics for patient 5 achieved both prior to reirradiation and after reirradiation using the coplanar and non-coplanar plans. Percentage differences in dosimetric values between the coplanar and non-coplanar reirradiation plans are reported for each parameter.

| **OAR** | **EQD2 parameter** | **Previous treatment(s)** | **Coplanar reirradiation plan** | **Non-coplanar reirradiation plan** |
| --- | --- | --- | --- | --- |
| Bronchial tree | D_max_ (Gy) | 66.0 | 66.0 | 66.0 (=) |
|  | D_mean_ (Gy) | 60.7 | 60.7 | 60.7 (=) |
| Esophagus | D_max_ (Gy) | 68.8 | 68.8 | 68.8 (=) |
|  | D_mean_ (Gy) | 32.2 | 32.3 | 32.4 (+0.3%) |
| Heart | D_max_ (Gy) | 64.4 | 64.4 | 64.4 (=) |
|  | D_mean_ (Gy) | 15.4 | 15.4 | 15.4 (=) |
| Spinal cord | D_max_ (Gy) | 53.8 | 53.8 | 53.8 (=) |
|  | D_mean_ (Gy) | 10.3 | 10.4 | 10.4 (=) |
| Trachea | D_max_ (Gy) | 66.7 | 66.7 | 66.7 (=) |
|  | D_mean_ (Gy) | 34.9 | 35.1 | 35.0 (-0.1%) |
| Lungs-GTV | D_mean_ (Gy) | 17.5 | 17.9 | 17.8 (-0.6%) |
|  | V_5Gy_ (%) | 89.3 | 90.5 | 90.2 (-0.3%) |
|  | V_20Gy_ (%) | 27.9 | 28.7 | 28.5 (-0.7%) |
| PTV | HI | - | 0.44 | 0.44 (=) |
|  | CI | - | 0.88 | 0.89 (+1.1) |

## C.6 Patient 6

The dose distributions along with the corresponding gantry-couch paths obtained for patient 6 are reported in Figure S5 for both the coplanar and non-coplanar reirradiation plans. The dosimetric results are instead detailed in Table S7.


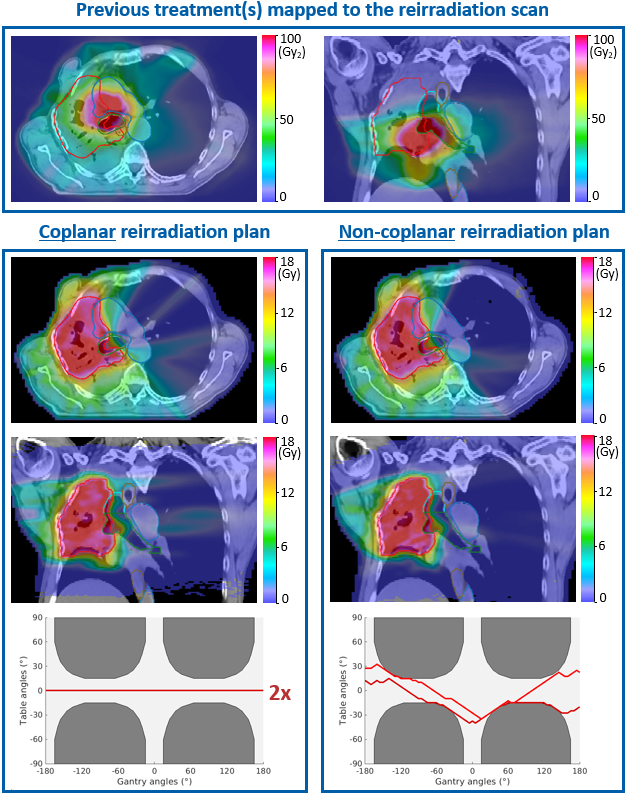


Figure S5: Comparison of the coplanar and non-coplanar reirradiation plans generated for patient 6. Contours of PTV (red), bronchial tree (green), esophagus (brown) and great vessel (blue) are delineated in both the transversal and coronal planes of the reirradiation scan. The gantry-couch paths for both reirradiation plans are also shown, where dark grey regions indicate beam orientations leading to collision between gantry and couch (and are therefore excluded from the set of candidate beam orientations).

Table S7: Cumulative EQD2 metrics for patient 6 achieved both prior to reirradiation and after reirradiation using the coplanar and non-coplanar plans. Percentage differences in dosimetric values between the coplanar and non-coplanar reirradiation plans are reported for each parameter.

| **OAR** | **EQD2 parameter** | **Previous treatment(s)** | **Coplanar reirradiation plan** | **Non-coplanar reirradiation plan** |
| --- | --- | --- | --- | --- |
| Bronchial tree^#^ | D_max_ (Gy) | 131.3 | 142.4 | 142.7 (+0.2%) |
|  | D_mean_ (Gy) | 68.5 | 72.5 | 71.5 (-1.4%) |
| Esophagus^#^ | D_max_ (Gy) | 92.4 | 99.5 | 95.8 (-3.7%) |
|  | D_mean_ (Gy) | 19.6 | 20.6 | 20.0 (-2.9%) |
| Heart^#^ | D_max_ (Gy) | 144.7 | 159.2 | 157.4 (-1.1%) |
|  | D_mean_ (Gy) | 12.9 | 13.7 | 13.7 (=) |
| Spinal cord | D_max_ (Gy) | 26.5 | 29.9 | 28.1 (-6.0%) |
|  | D_mean_ (Gy) | 2.69 | 3.65 | 3.54 (-3.0%) |
| Great vessel^#^ | D_max_ (Gy) | 143.5 | 159.6 | 158.5 (-0.7%) |
|  | D_mean_ (Gy) | 41.0 | 43.4 | 42.8 (-1.4%) |
| Trachea^#^ | D_max_ (Gy) | 49.6 | 52.7 | 50.0 (-5.1%) |
|  | D_mean_ (Gy) | 9.29 | 11.2 | 10.2 (-8.9%) |
| Lungs-GTV | D_mean_ (Gy) | 9.22 | 10.1 | 9.88 (-2.2%) |
|  | V_5Gy_ (%) | 53.5 | 54.3 | 53.9 (-0.7%) |
|  | V_20Gy_ (%) | 14.4 | 14.9 | 14.4 (-0.5%) |
| PTV | HI | - | 0.22 | 0.27 (+18.5%) |
|  | CI | - | 0.27 | 0.32 (+22.7%) |

^#^ OARs which overlap with the PTV

## C.7 Patient 7

The dose distributions along with the corresponding gantry-couch paths obtained for patient 7 are reported in Figure S6 for both the coplanar and non-coplanar reirradiation plans. The dosimetric results are instead detailed in Table S8.


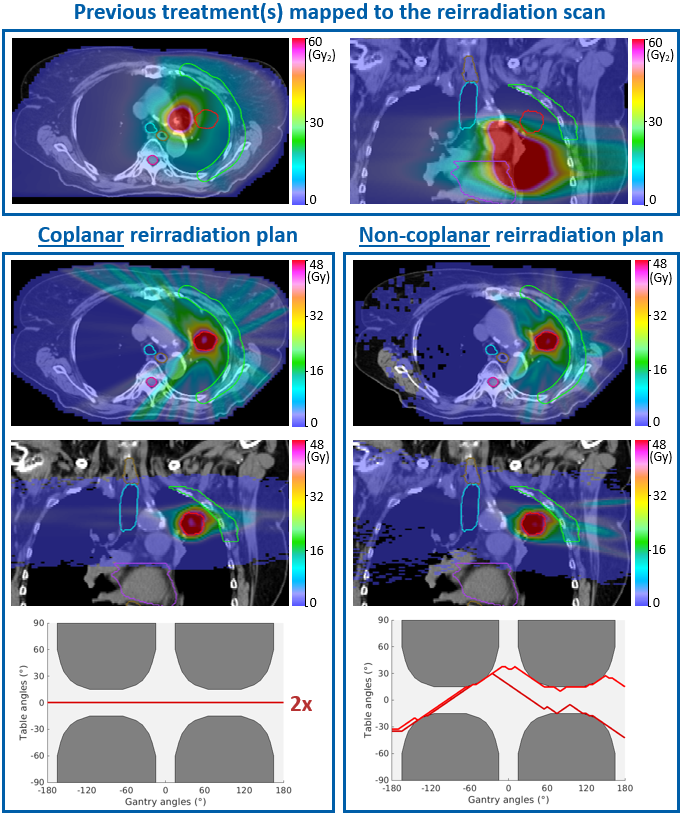


Figure S6: Comparison of the coplanar and non-coplanar reirradiation plans generated for patient 7. Contours of PTV (red), thoracic wall (light green), trachea (light blue), heart (violet) and spinal cord (pink) are delineated in both the transversal and coronal planes of the reirradiation scan. The gantry-couch paths for both reirradiation plans are also shown, where dark grey regions indicate beam orientations leading to collision between gantry and couch (and are therefore excluded from the set of candidate beam orientations).

Table S8: Cumulative EQD2 metrics for patient 7 achieved both prior to reirradiation and after reirradiation using the coplanar and non-coplanar plans. Percentage differences in dosimetric values between the coplanar and non-coplanar reirradiation plans are reported for each parameter.

| **OAR** | **EQD2 parameter** | **Previous treatment(s)** | **Coplanar reirradiation plan** | **Non-coplanar reirradiation plan** |
| --- | --- | --- | --- | --- |
| Esophagus | D_max_ (Gy) | 28.2 | 28.2 | 28.2 (=) |
|  | D_mean_ (Gy) | 7.45 | 7.64 | 7.62 (-0.3%) |
| Heart | D_max_ (Gy) | 72.0 | 72.0 | 72.0 (=) |
|  | D_mean_ (Gy) | 14.2 | 14.2 | 14.2 (=) |
| Spinal cord | D_max_ (Gy) | 9.46 | 10.9 | 9.47 (-13.1%) |
|  | D_mean_ (Gy) | 1.77 | 1.93 | 1.96 (+1.6%) |
| Thoracic wall^#^ | D_max_ (Gy) | 27.1 | 98.7 | 91.2 (-7.6%) |
|  | D_mean_ (Gy) | 5.61 | 14.2 | 12.4 (-12.7%) |
| Trachea | D_max_ (Gy) | 14.7 | 15.2 | 14.8 (-2.6%) |
|  | D_mean_ (Gy) | 2.12 | 2.47 | 2.27 (-8.1%) |
| Lungs-GTV | D_mean_ (Gy) | 8.57 | 11.2 | 11.0 (-1.8%) |
|  | V_5Gy_ (%) | 32.7 | 35.9 | 34.6 (-3.6%) |
|  | V_20Gy_ (%) | 12.5 | 16.3 | 15.9 (-2.5%) |
| PTV | HI | - | 0.31 | 0.31 (=) |
|  | CI | - | 0.42 | 0.43 (+2.4%) |

^#^ OARs which overlap with the PTV

## C.8 Patient 8

The dose distributions along with the corresponding gantry-couch paths obtained for patient 8 are reported in Figure S7 for both the coplanar and non-coplanar reirradiation plans. The dosimetric results are instead detailed in Table S9.


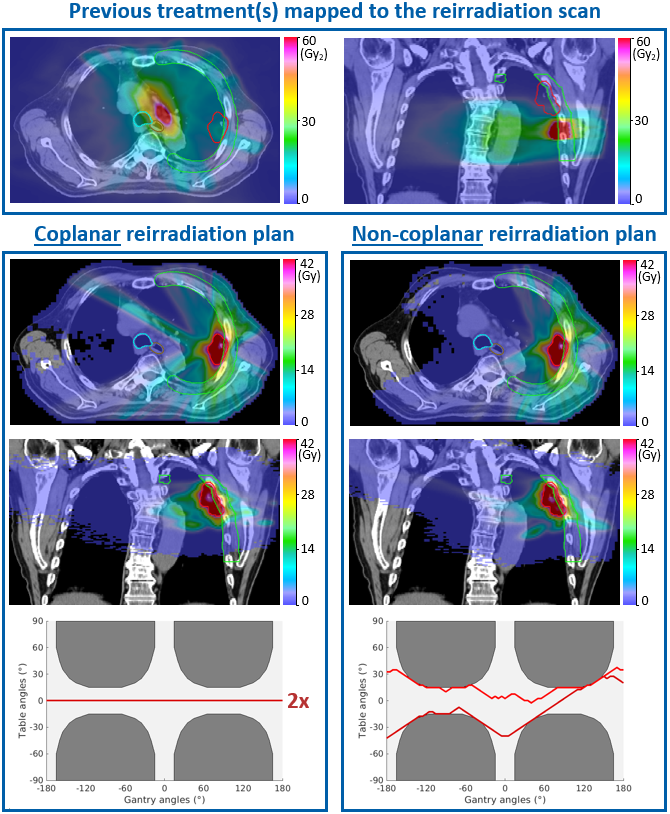


Figure S7: Comparison of the coplanar and non-coplanar reirradiation plans generated for patient 8. Contours of PTV (red), thoracic wall (light green), trachea (light blue) and esophagus (brown) are delineated in both the transversal and coronal planes of the reirradiation scan. The gantry-couch paths for both reirradiation plans are also shown, where dark grey regions indicate beam orientations leading to collision between gantry and couch (and are therefore excluded from the set of candidate beam orientations).

Table S9: Cumulative EQD2 metrics for patient 8 achieved both prior to reirradiation and after reirradiation using the coplanar and non-coplanar plans. Percentage differences in dosimetric values between the coplanar and non-coplanar reirradiation plans are reported for each parameter.

| **OAR** | **EQD2 parameter** | **Previous treatment(s)** | **Coplanar reirradiation plan** | **Non-coplanar reirradiation plan** |
| --- | --- | --- | --- | --- |
| Bronchial tree | D_max_ (Gy) | 56.9 | 56.9 | 56.9 (=) |
|  | D_mean_ (Gy) | 33.1 | 33.1 | 33.1 (=) |
| Esophagus | D_max_ (Gy) | 45.8 | 45.8 | 45.8 (=) |
|  | D_mean_ (Gy) | 15.3 | 15.3 | 15.5 (+1.3%) |
| Heart | D_max_ (Gy) | 64.1 | 64.1 | 64.1 (=) |
|  | D_mean_ (Gy) | 5.08 | 5.08 | 5.08 (=) |
| Spinal cord | D_max_ (Gy) | 12.8 | 14.8 | 13.5 (-1.3%) |
|  | D_mean_ (Gy) | 1.95 | 2.21 | 2.12 (-0.1%) |
| Thoracic wall^#^ | D_max_ (Gy) | 237.8 | 270.9 | 270.3 (-0.2%) |
|  | D_mean_ (Gy) | 12.3 | 18.7 | 18.5 (-1.1%) |
| Trachea | D_max_ (Gy) | 44.4 | 44.4 | 44.6 (+0.5%) |
|  | D_mean_ (Gy) | 12.5 | 12.6 | 12.8 (+1.6%) |
| Lungs-GTV | D_mean_ (Gy) | 7.02 | 8.72 | 8.63 (-1.0%) |
|  | V_5Gy_ (%) | 40.5 | 42.8 | 42.7 (-0.2%) |
|  | V_20Gy_ (%) | 7.28 | 10.7 | 10.2 (-4.7%) |
| PTV | HI | - | 0.27 | 0.24 (-11.1%) |
|  | CI | - | 0.53 | 0.53 (=) |

^#^ OARs which overlap with the PTV

## C.9 Patient 9

The dose distributions along with the corresponding gantry-couch paths obtained for patient 9 are reported in Figure S8 for both the coplanar and non-coplanar reirradiation plans. The dosimetric results are instead detailed in Table S10.


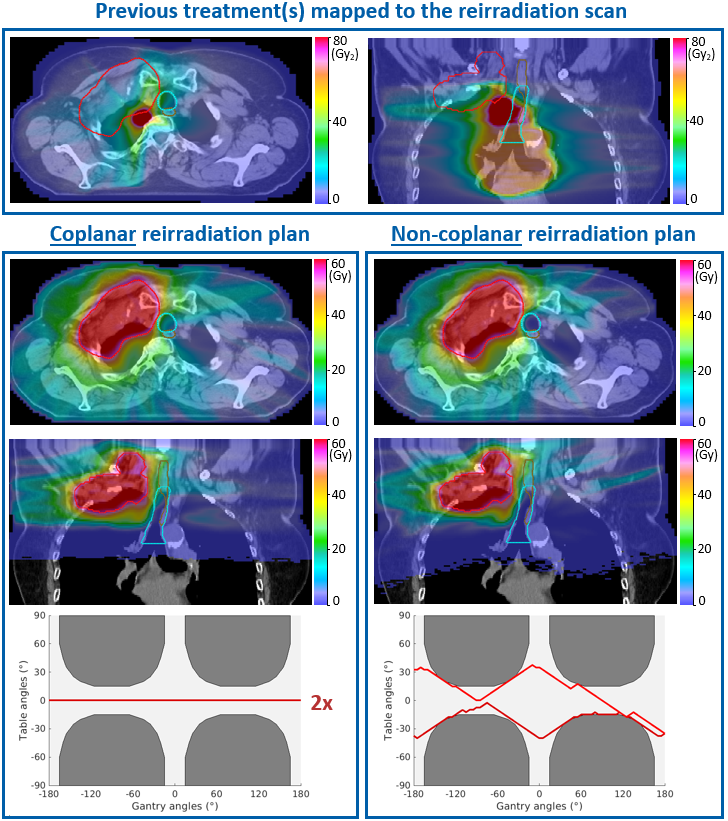


Figure S8: Comparison of the coplanar and non-coplanar reirradiation plans generated for patient 9. Contours of PTV (red), trachea (light blue) and esophagus (brown) are delineated in both the transversal and coronal planes of the reirradiation scan. The gantry-couch paths for both reirradiation plans are also shown, where dark grey regions indicate beam orientations leading to collision between gantry and couch (and are therefore excluded from the set of candidate beam orientations).

Table S10: Cumulative EQD2 metrics for patient 9 achieved both prior to reirradiation and after reirradiation using the coplanar and non-coplanar plans. Percentage differences in dosimetric values between the coplanar and non-coplanar reirradiation plans are reported for each parameter.

| **OAR** | **EQD2 parameter** | **Previous treatment(s)** | **Coplanar reirradiation plan** | **Non-coplanar reirradiation plan** |
| --- | --- | --- | --- | --- |
| Esophagus | D_max_ (Gy) | 83.6 | 84.8 | 83.7 (-1.3%) |
|  | D_mean_ (Gy) | 33.3 | 35.4 | 34.8 (-1.7%) |
| Heart | D_max_ (Gy) | 66.7 | 66.7 | 66.7 (=) |
|  | D_mean_ (Gy) | 7.67 | 7.67 | 7.67 (=) |
| Spinal cord | D_max_ (Gy) | 33.2 | 37.1 | 33.5 (-9.7%) |
|  | D_mean_ (Gy) | 7.51 | 9.33 | 90.5 (-3.0%) |
| Right brachial plexus^#^ | D_max_ (Gy) | 3.47 | 50.3 | 44.6 (-11.3%) |
|  | D_mean_ (Gy) | 1.40 | 19.2 | 12.9 (-32.8%) |
| Trachea^#^ | D_max_ (Gy) | 91.1 | 102.7 | 97.4 (-5.2%) |
|  | D_mean_ (Gy) | 40.2 | 46.0 | 42.5 (-7.6%) |
| Lungs-GTV | D_mean_ (Gy) | 12.2 | 13.4 | 13.2 (-1.5%) |
|  | V_5Gy_ (%) | 57.3 | 57.4 | 57.4 (=) |
|  | V_20Gy_ (%) | 21.3 | 22.7 | 22.5 (-0.9%) |
| PTV | HI | - | 0.14 | 0.15 (+7.1%) |
|  | CI | - | 0.49 | 0.54 (+10.2%) |

^#^ OARs which overlap with the PTV

## C.10 Patient 10

The dose distributions along with the corresponding gantry-couch paths obtained for patient 10 are reported in Figure S9 for both the coplanar and non-coplanar reirradiation plans. The dosimetric results are instead detailed in Table S11.


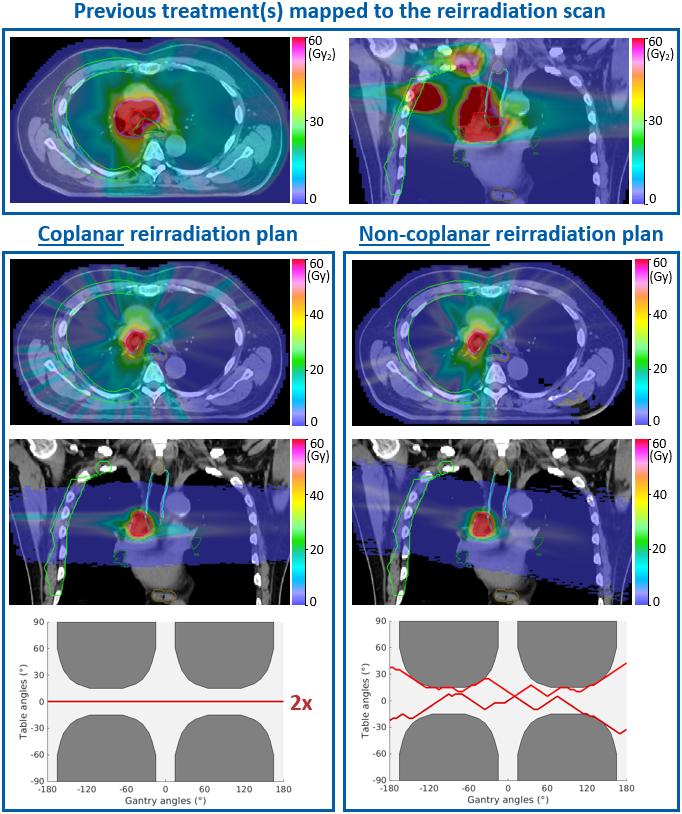


Figure S9: Comparison of the coplanar and non-coplanar reirradiation plans generated for patient 10. Contours of PTV (red), trachea (light blue), bronchial tree (dark green), thoracic wall (light green) and esophagus (brown) are delineated in both the transversal and coronal planes of the reirradiation scan. The gantry-couch paths for both reirradiation plans are also shown, where dark grey regions indicate beam orientations leading to collision between gantry and couch (and are therefore excluded from the set of candidate beam orientations).

Table S11: Cumulative EQD2 metrics for patient 10 achieved both prior to reirradiation and after reirradiation using the coplanar and non-coplanar plans. Percentage differences in dosimetric values between the coplanar and non-coplanar reirradiation plans are reported for each parameter.

| **OAR** | **EQD2 parameter** | **Previous treatment(s)** | **Coplanar reirradiation plan** | **Non-coplanar reirradiation plan** |
| --- | --- | --- | --- | --- |
| Bronchial tree^#^ | D_max_ (Gy) | 70.4 | 121.2 | 121.2 (=) |
|  | D_mean_ (Gy) | 24.3 | 31.1 | 30.1 (-3.2%) |
| Esophagus | D_max_ (Gy) | 65.1 | 65.2 | 65.2 (=) |
|  | D_mean_ (Gy) | 7.90 | 7.95 | 7.94 (-0.1%) |
| Heart | D_max_ (Gy) | 1.30 | 1.31 | 1.31 (=) |
|  | D_mean_ (Gy) | 0.45 | 0.45 | 0.45 (=) |
| Spinal cord | D_max_ (Gy) | 50.0 | 50.6 | 51.0 (+0.8%) |
|  | D_mean_ (Gy) | 5.46 | 6.02 | 6.09 (+1.2%) |
| Thoracic wall | D_max_ (Gy) | 173.9 | 173.9 | 173.9 (=) |
|  | D_mean_ (Gy) | 11.2 | 12.2 | 12.1 (-0.8%) |
| Trachea^#^ | D_max_ (Gy) | 71.3 | 115.1 | 111.4 (-3.2%) |
|  | D_mean_ (Gy) | 24.2 | 25.5 | 25.1 (-1.6%) |
| Lungs-GTV | D_mean_ (Gy) | 8.25 | 9.16 | 9.05 (-1.2%) |
|  | V_5Gy_ (%) | 35.5 | 35.9 | 35.9 (=) |
|  | V_20Gy_ (%) | 11.1 | 13.5 | 12.8 (-5.2%) |
| PTV | HI | - | 0.07 | 0.09 (+28.6%) |
|  | CI | - | 0.48 | 0.47 (-2.1%) |

^#^ OARs which overlap with the PTV

## C.11 Patient 11

The dose distributions along with the corresponding gantry-couch paths obtained for patient 11 are reported in Figure S10 for both the coplanar and non-coplanar reirradiation plans. The dosimetric results are instead detailed in Table S12.


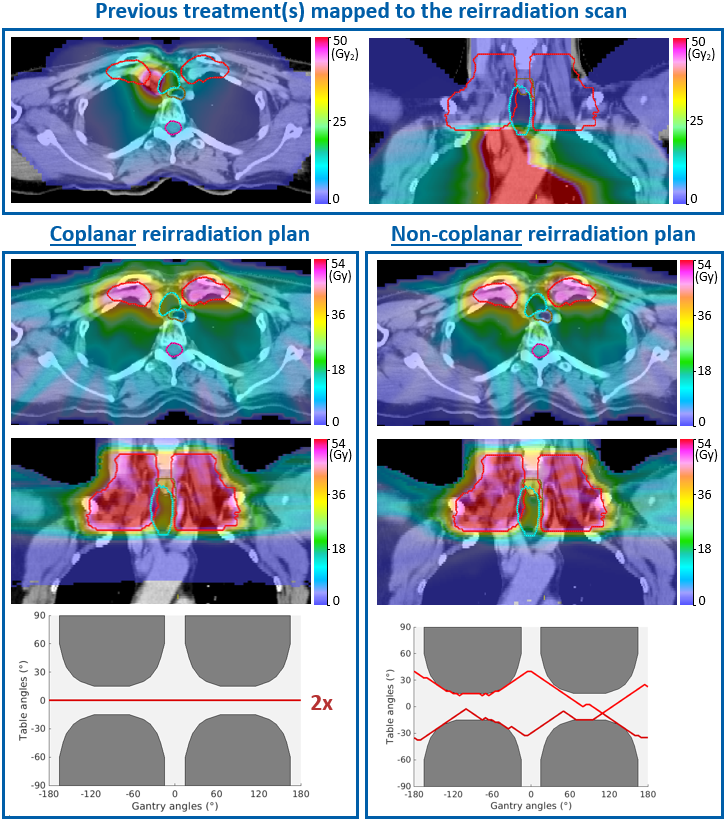


Figure S10: Comparison of the coplanar and non-coplanar reirradiation plans generated for patient 11. Contours of PTV (red), trachea (light blue), spinal cord (pink) and esophagus (brown) are delineated in both the transversal and coronal planes of the reirradiation scan. The gantry-couch paths for both reirradiation plans are also shown, where dark grey regions indicate beam orientations leading to collision between gantry and couch (and are therefore excluded from the set of candidate beam orientations).

Table S12: Cumulative EQD2 metrics for patient 11 achieved both prior to reirradiation and after reirradiation using the coplanar and non-coplanar plans. Percentage differences in dosimetric values between the coplanar and non-coplanar reirradiation plans are reported for each parameter.

| **OAR** | **EQD2 parameter** | **Previous treatment(s)** | **Coplanar reirradiation plan** | **Non-coplanar reirradiation plan** |
| --- | --- | --- | --- | --- |
| Esophagus^#^ | D_max_ (Gy) | 69.4 | 69.4 | 69.4 (=) |
|  | D_mean_ (Gy) | 25.0 | 36.7 | 32.7 (-10.9%) |
| Spinal cord | D_max_ (Gy) | 19.0 | 19.5 | 20.7 (+6.2%) |
|  | D_mean_ (Gy) | 4.35 | 7.98 | 7.44 (-6.8%) |
| Thyroid^#^ | D_max_ (Gy) | 0.41 | 39.4 | 30.9 (-21.6%) |
|  | D_mean_ (Gy) | 0.27 | 12.3 | 7.34 (-40.3%) |
| Trachea^#^ | D_max_ (Gy) | 69.9 | 84.6 | 79.5 (-6.0%) |
|  | D_mean_ (Gy) | 18.8 | 35.4 | 30.8 (-13.0%) |
| Right brachial plexus^#^ | D_max_ (Gy) | 0.64 | 31.9 | 22.5 (-29.5%) |
|  | D_mean_ (Gy) | 0.58 | 14.2 | 7.1 (-50.0%) |
| Left brachial plexus^#^ | D_max_ (Gy) | 0.39 | 27.9 | 21.5 (-1.2%) |
|  | D_mean_ (Gy) | 0.33 | 10.1 | 5.57 (-44.9) |
| PTV | HI | - | 0.24 | 0.29 (+20.8%) |
|  | CI | - | 0.75 | 0.74 (-1.3%) |

^#^ OARs which overlap with the PTV

## C.12 Patient 12

The dose distributions along with the corresponding gantry-couch paths obtained for patient 12 are reported in Figure S11 for both the coplanar and non-coplanar reirradiation plans. The dosimetric results are instead detailed in Table S13.


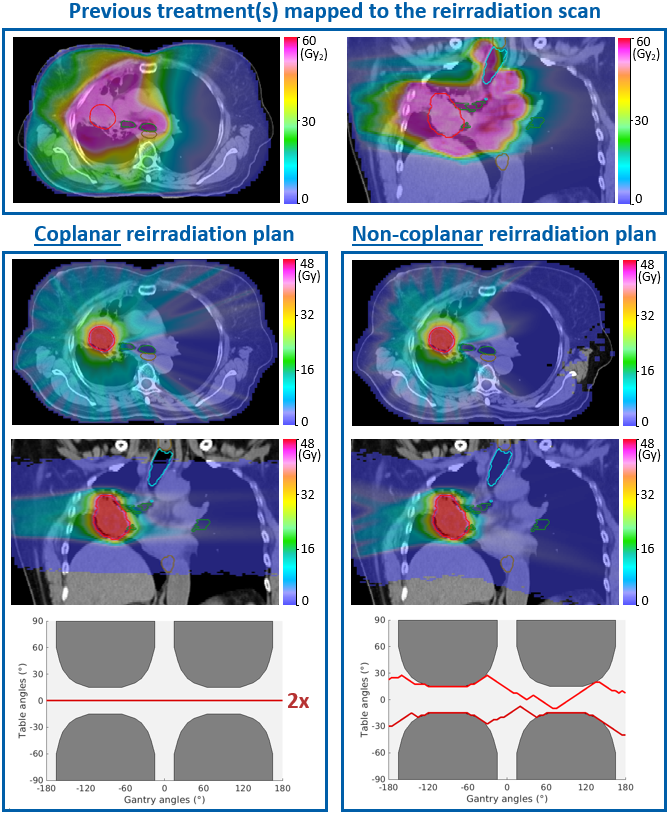


Figure S11: Comparison of the coplanar and non-coplanar reirradiation plans generated for patient 12. Contours of PTV (red), trachea (light blue), bronchial tree (green) and esophagus (brown) are delineated in both the transversal and coronal planes of the reirradiation scan. The gantry-couch paths for both reirradiation plans are also shown, where dark grey regions indicate beam orientations leading to collision between gantry and couch (and are therefore excluded from the set of candidate beam orientations).

Table S13: Cumulative EQD2 metrics for patient 12 achieved both prior to reirradiation and after reirradiation using the coplanar and non-coplanar plans. Percentage differences in dosimetric values between the coplanar and non-coplanar reirradiation plans are reported for each parameter.

| **OAR** | **EQD2 parameter** | **Previous treatment(s)** | **Coplanar reirradiation plan** | **Non-coplanar reirradiation plan** |
| --- | --- | --- | --- | --- |
| Bronchial tree^#^ | D_max_ (Gy) | 58.2 | 112.3 | 104.1 (-7.3%) |
|  | D_mean_ (Gy) | 45.5 | 51.3 | 49.0 (-4.5%) |
| Esophagus | D_max_ (Gy) | 58.1 | 58.8 | 58.3 (-0.9%) |
|  | D_mean_ (Gy) | 28.2 | 28.5 | 28.3 (-0.7%) |
| Heart | D_max_ (Gy) | 57.3 | 64.1 | 63.4 (-1.1%) |
|  | D_mean_ (Gy) | 9.92 | 10.2 | 10.4 (+2.0%) |
| Spinal cord | D_max_ (Gy) | 26.5 | 28.3 | 27.4 (-3.2%) |
|  | D_mean_ (Gy) | 7.88 | 8.31 | 8.36 (+0.6%) |
| Trachea | D_max_ (Gy) | 58.3 | 59.7 | 58.3 (-2.3%) |
|  | D_mean_ (Gy) | 33.5 | 33.6 | 33.6 (=) |
| Liver-GTV | D_max_ (Gy) | 57.9 | 111.1 | 108.5 (-2.3%) |
|  | D_mean_ (Gy) | 4.41 | 4.54 | 4.53 (-0.2%) |
| Lungs-GTV | D_mean_ (Gy) | 14.7 | 17.1 | 16.8 (-1.8%) |
|  | V_5Gy_ (%) | 55.2 | 59.9 | 56.4 (-5.8%) |
|  | V_20Gy_ (%) | 24.5 | 25.4 | 25.3 (-0.4%) |
| PTV | HI | - | 0.13 | 0.13 (=) |
|  | CI | - | 0.48 | 0.46 (-4.2%) |

^#^ OARs which overlap with the PTV

## C.13 Patient 13

The dose distributions along with the corresponding gantry-couch paths obtained for patient 13 are reported in Figure S12 for both the coplanar and non-coplanar reirradiation plans. The dosimetric results are instead detailed in Table S14.


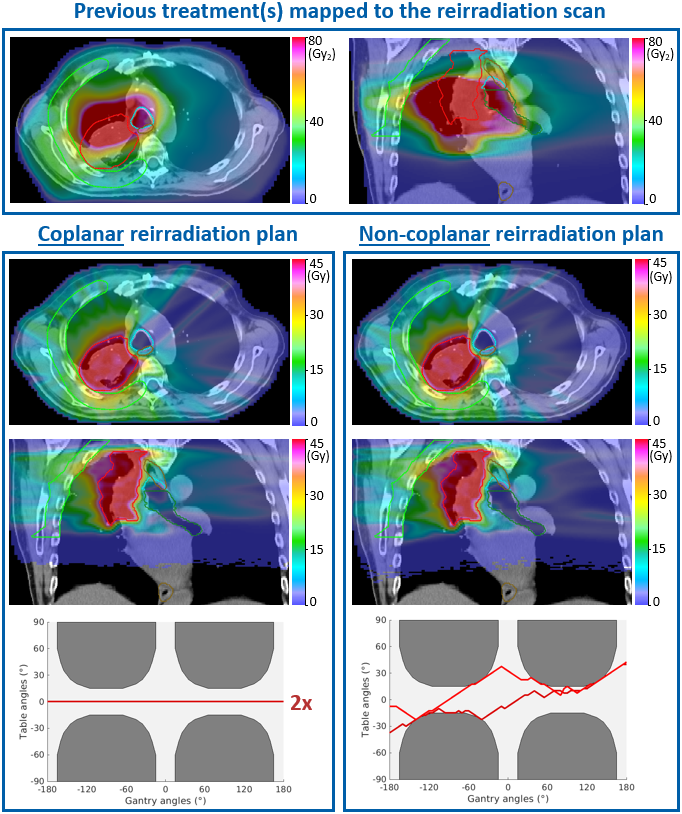


Figure S12: Comparison of the coplanar and non-coplanar reirradiation plans generated for patient 13. Contours of PTV (red), trachea (light blue), bronchial tree (dark green), thoracic wall (light green) and esophagus (brown) are delineated in both the transversal and coronal planes of the reirradiation scan. The gantry-couch paths for both reirradiation plans are also shown, where dark grey regions indicate beam orientations leading to collision between gantry and couch (and are therefore excluded from the set of candidate beam orientations).

Table S14: Cumulative EQD2 metrics for patient 13 achieved both prior to reirradiation and after reirradiation using the coplanar and non-coplanar plans. Percentage differences in dosimetric values between the coplanar and non-coplanar reirradiation plans are reported for each parameter.

| **OAR** | **EQD2 parameter** | **Previous treatment(s)** | **Coplanar reirradiation plan** | **Non-coplanar reirradiation plan** |
| --- | --- | --- | --- | --- |
| Bronchial tree^#^ | D_max_ (Gy) | 127.4 | 129.2 | 12.90 (-0.2%) |
|  | D_mean_ (Gy) | 65.7 | 68.5 | 67.9 (-0.9%) |
| Esophagus^#^ | D_max_ (Gy) | 79.2 | 81.4 | 81.1 (-0.4%) |
|  | D_mean_ (Gy) | 29.6 | 31.5 | 31.6 (+0.3%) |
| Heart | D_max_ (Gy) | 66.6 | 66.8 | 66.7 (-0.1%) |
|  | D_mean_ (Gy) | 4.77 | 4.78 | 4.80 (+0.4%) |
| Spinal cord | D_max_ (Gy) | 24.9 | 30.4 | 28.6 (-5.9%) |
|  | D_mean_ (Gy) | 4.83 | 6.54 | 6.58 (+0.6%) |
| Thoracic wall^#^ | D_max_ (Gy) | 67.5 | 88.7 | 86.6 (-2.4%) |
|  | D_mean_ (Gy) | 21.5 | 35.2 | 34.9 (-0.9%) |
| Trachea^#^ | D_max_ (Gy) | 89.4 | 92.6 | 91.0 (-1.7%) |
|  | D_mean_ (Gy) | 47.3 | 49.8 | 49.0 (-1.6%) |
| Right brachial plexus | D_max_ (Gy) | 6.89 | 12.8 | 13.4 (+4.7%) |
|  | D_mean_ (Gy) | 2.17 | 3.29 | 4.10 (+24.6%) |
| Lungs-GTV | D_mean_ (Gy) | 17.0 | 21.7 | 21.6 (-0.5%) |
|  | V_5Gy_ (%) | 64.3 | 64.8 | 65.1 (+0.5%) |
|  | V_20Gy_ (%) | 26.8 | 31.7 | 32.1 (-1.6%) |
| PTV | HI | - | 0.20 | 0.19 (-5.0%) |
|  | CI | - | 0.41 | 0.42 (+2.4%) |

^#^ OARs which overlap with the PTV

## C.14 Patient 14

The dose distributions along with the corresponding gantry-couch paths obtained for patient 14 are reported in Figure S13 for both the coplanar and non-coplanar reirradiation plans. The dosimetric results are instead detailed in Table S15.


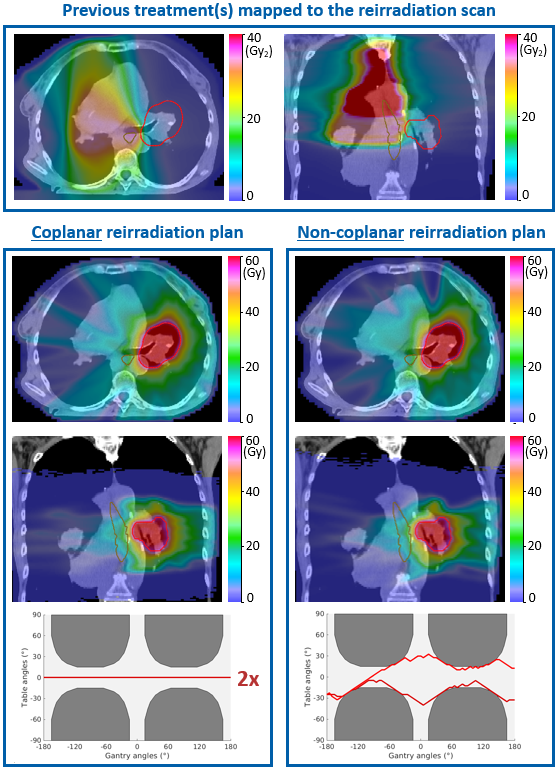


Figure S13: Comparison of the coplanar and non-coplanar reirradiation plans generated for patient 14. Contours of PTV (red) and esophagus (brown) are delineated in both the transversal and coronal planes of the reirradiation scan. The gantry-couch paths for both reirradiation plans are also shown, where dark grey regions indicate beam orientations leading to collision between gantry and couch (and are therefore excluded from the set of candidate beam orientations).

Table S15: Cumulative EQD2 metrics for patient 14 achieved both prior to reirradiation and after reirradiation using the coplanar and non-coplanar plans. Percentage differences in dosimetric values between the coplanar and non-coplanar reirradiation plans are reported for each parameter.

| **OAR** | **EQD2 parameter** | **Previous treatment(s)** | **Coplanar reirradiation plan** | **Non-coplanar reirradiation plan** |
| --- | --- | --- | --- | --- |
| Esophagus | D_max_ (Gy) | 63.0 | 63.0 | 63.0 (=) |
|  | D_mean_ (Gy) | 30.1 | 33.3 | 33.3 (=) |
| Heart^#^ | D_max_ (Gy) | 35.3 | 67.4 | 66.5 (-1.3%) |
|  | D_mean_ (Gy) | 3.18 | 7.32 | 6.49 (-11.3%) |
| Lungs-GTV | D_mean_ (Gy) | 7.92 | 13.2 | 12.8 (-3.0%) |
|  | V_5Gy_ (%) | 45.3 | 64.9 | 63.9 (-1.5%) |
|  | V_20Gy_ (%) | 12.7 | 23.4 | 21.7 (-7.3%) |
| PTV | HI | - | 0.09 | 0.11 (+22.2%) |
|  | CI | - | 0.48 | 0.48 (=) |

^#^ OARs which overlap with the PTV

## C.15 Patient 15

The dose distributions along with the corresponding gantry-couch paths obtained for patient 15 are reported in Figure S14 for both the coplanar and non-coplanar reirradiation plans. The dosimetric results are instead detailed in Table S16.


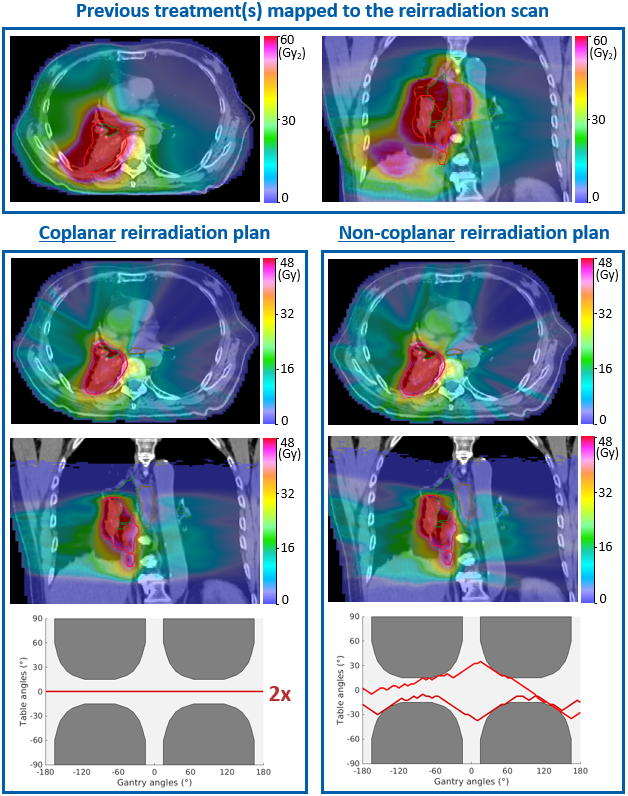


Figure S14: Comparison of the coplanar and non-coplanar reirradiation plans generated for patient 15. Contours of PTV (red), bronchial tree (green) and esophagus (brown) are delineated in both the transversal and coronal planes of the reirradiation scan. The gantry-couch paths for both reirradiation plans are also shown, where dark grey regions indicate beam orientations leading to collision between gantry and couch (and are therefore excluded from the set of candidate beam orientations).

Table S16: Cumulative EQD2 metrics for patient 15 achieved both prior to reirradiation and after reirradiation using the coplanar and non-coplanar plans. Percentage differences in dosimetric values between the coplanar and non-coplanar reirradiation plans are reported for each parameter.

| **OAR** | **EQD2 parameter** | **Previous treatment(s)** | **Coplanar reirradiation plan** | **Non-coplanar reirradiation plan** |
| --- | --- | --- | --- | --- |
| Bronchial tree^#^ | D_max_ (Gy) | 64.6 | 113.0 | 114.0 (+0.9%) |
|  | D_mean_ (Gy) | 51.6 | 55.1 | 54.8 (-0.5%) |
| Esophagus | D_max_ (Gy) | 64.4 | 64.8 | 64.7 (-0.2%) |
|  | D_mean_ (Gy) | 21.9 | 23.0 | 23.3 (+1.3%) |
| Heart^#^ | D_max_ (Gy) | 60.6 | 89.4 | 89.1 (-0.3%) |
|  | D_mean_ (Gy) | 5.10 | 9.82 | 9.56 (-2.6%) |
| Spinal cord | D_max_ (Gy) | 42.5 | 51.6 | 51.8 (+0.4%) |
|  | D_mean_ (Gy) | 12.9 | 14.6 | 14.7 (+0.7%) |
| Trachea | D_max_ (Gy) | 65.3 | 65.4 | 65.4 (=) |
|  | D_mean_ (Gy) | 27.8 | 27.8 | 27.8 (=) |
| Lungs-GTV | D_mean_ (Gy) | 12.7 | 15.8 | 15.7 (-0.6%) |
|  | V_5Gy_ (%) | 74.7 | 78.0 | 77.6 (-0.5%) |
|  | V_20Gy_ (%) | 17.8 | 22.9 | 22.3 (-2.6%) |
| PTV | HI | - | 0.18 | 0.17 (-5.6%) |
|  | CI | - | 0.43 | 0.43 (=) |

^#^ OARs which overlap with the PTV

## C.16 All patients

The cumulative EQD2 delivered to the most critical thoracic OARs by both the coplanar and non-colpanar reirradiation plans are summarized for all patients in Figure S15.


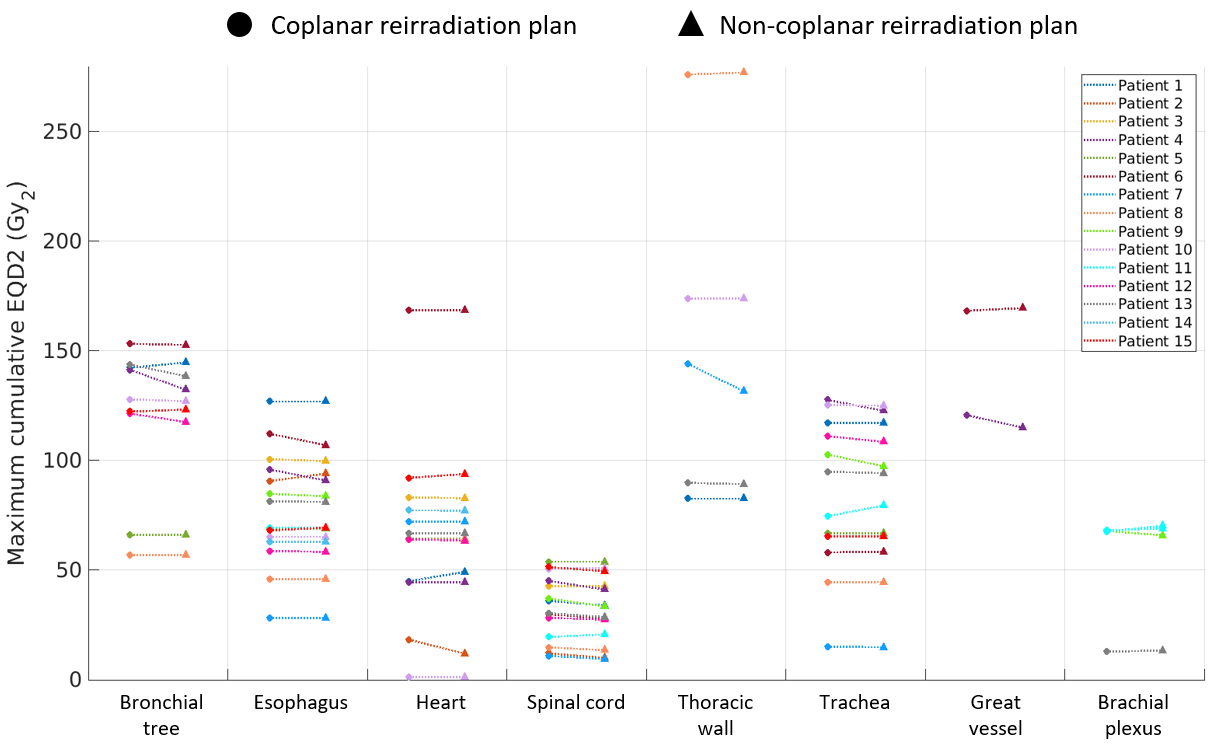


Figure S15: Comparison of the maximum cumulative EQD2 delivered to the most critical thoracic OARs in all patients using coplanar (round marker) and non-coplanar (triangular marker) reirradiation plans, respectively.
